# Clonal driver neoantigen loss under *EGFR* TKI and immune selection pressures

**DOI:** 10.1038/s41586-025-08586-y

**Published:** 2025-02-19

**Authors:** Maise Al Bakir, James L. Reading, Samuel Gamble, Rachel Rosenthal, Imran Uddin, Andrew Rowan, Joanna Przewrocka, Amber Rogers, Yien Ning Sophia Wong, Amalie K. Bentzen, Selvaraju Veeriah, Sophia Ward, Aaron T. Garnett, Paula Kalavakur, Carlos Martínez-Ruiz, Clare Puttick, Ariana Huebner, Daniel E. Cook, David A. Moore, Chris Abbosh, Crispin T. Hiley, Cristina Naceur-Lombardelli, Thomas B. K. Watkins, Marina Petkovic, Roland F. Schwarz, Felipe Gálvez-Cancino, Kevin Litchfield, Peter Meldgaard, Boe Sandahl Sorensen, Line Bille Madsen, Dirk Jäger, Martin D. Forster, Tobias Arkenau, Clara Domingo-Vila, Timothy I. M. Tree, Mohammad Kadivar, Sine Reker Hadrup, Benny Chain, Sergio A. Quezada, Nicholas McGranahan, Charles Swanton

**Affiliations:** 1 Cancer Evolution and Genome Instability Laboratory, The Francis Crick Institute, London, UK; 2 Cancer Research UK Lung Cancer Centre of Excellence, University College London Cancer Institute, London, UK; 3 Pre-Cancer Immunology Laboratory, Research Department of Haematology, University College London Cancer Institute, London, UK; 4 Division of Infection and Immunity, University College London, London, UK; 5 Genomics Science Technology Platform, The Francis Crick Institute, London, UK; 6 Invitae Inc., Boulder, CO USA; 7 Cancer Genome Evolution Research Group, University College London Cancer Institute, University College London, London, UK; 8 Department of Cellular Pathology, University College London Hospital NHS Foundation Trust, London, UK; 9 Berlin Institute for Medical Systems Biology, Max Delbrück Center for Molecular Medicine in the Helmholtz Association, Berlin, Germany; 10 Department of Biology, Humboldt University of Berlin, Berlin, Germany; 11 Division of Oncology and Hematology, Department of Pediatrics, Charité – Universitätsmedizin Berlin, Corporate Member of Freie Universität Berlin and Humboldt Universität zu Berlin, Berlin, Germany; 12 Institute for Computational Cancer Biology (ICCB), Center for Integrated Oncology (CIO), Cancer Research Center Cologne Essen (CCCE), Faculty of Medicine and University Hospital Cologne, University of Cologne, Cologne, Germany; 13 Berlin Institute for the Foundations of Learning and Data (BIFOLD), Berlin, Germany; 14 Immune-Regulation and Immune-Interactions Laboratory, Centre for Immuno-Oncology, Nuffield Department of Medicine, University of Oxford, Headington, UK; 15 Department of Oncology, Aarhus University Hospital, Aarhus, Denmark; 16 Department of Clinical Biochemistry, Aarhus University Hospital, Aarhus, Denmark; 17 Department of Pathology, Aarhus University Hospital, Aarhus, Denmark; 18 Department of Medical Oncology, National Center for Tumor Diseases Heidelberg, Heidelberg University Hospital, Heidelberg, Germany; 19 Department of Oncology, UCL Cancer Institute, London, UK; 20 Sarah Cannon Research Institute, London, UK; 21 Department of Immunobiology, Faculty of Life Sciences and Medicine, King’s College London, London, UK; 22 Department of Health Technology, Technical University of Denmark, Lyngby, Denmark; 23 Department of Computer Sciences, University College London, London, UK; 24 Cancer Immunology Unit, Research Department of Haematology, University College London Cancer Institute, London, UK

**Keywords:** Non-small-cell lung cancer, Cancer genomics, Tumour immunology, Adaptive immunity

## Abstract

Neoantigen vaccines are under investigation for various cancers, including epidermal growth factor receptor (*EGFR*)-driven lung cancers^1,2^. We tracked the phylogenetic history of an *EGFR* mutant lung cancer treated with erlotinib, osimertinib, radiotherapy and a personalized neopeptide vaccine (NPV) targeting ten somatic mutations, including *EGFR* exon 19 deletion (ex19del). The ex19del mutation was clonal, but is likely to have appeared after a whole-genome doubling (WGD) event. Following osimertinib and NPV treatment, loss of the ex19del mutation was identified in a progressing small-cell-transformed liver metastasis. Circulating tumour DNA analyses tracking 467 somatic variants revealed the presence of this *EGFR* wild-type clone before vaccination and its expansion during osimertinib/NPV therapy. Despite systemic T cell reactivity to the vaccine-targeted ex19del neoantigen, the NPV failed to halt disease progression. The liver metastasis lost vaccine-targeted neoantigens through chromosomal instability and exhibited a hostile microenvironment, characterized by limited immune infiltration, low *CXCL9* and elevated M2 macrophage levels. Neoantigens arising post-WGD were more likely to be absent in the progressing liver metastasis than those occurring pre-WGD, suggesting that prioritizing pre-WGD neoantigens may improve vaccine design. Data from the TRACERx 421 cohort^3^ provide evidence that pre-WGD mutations better represent clonal variants, and owing to their presence at multiple copy numbers, are less likely to be lost in metastatic transition. These data highlight the power of phylogenetic disease tracking and functional T cell profiling to understand mechanisms of immune escape during combination therapies.

## Abstract (web summary)

We show the evolution of a case of *EGFR* mutant lung cancer treated with a combination of erlotinib, osimertinib, radiotherapy and a personalized neopeptide vaccine targeting somatic mutations, including *EGFR* exon 19 deletion.

## Main

Activating mutations of the epidermal growth factor receptor (*EGFR*) gene occur in approximately 10% of European/North American lung adenocarcinoma cases^1^, and up to 50–60% of cases in East Asia^4,5^. These mutations, particularly exon 19 deletion (ex19del) and L858R mutations in exon 21, confer sensitivity to *EGFR* tyrosine kinase inhibitors (TKIs) and are associated with improved outcomes. However, resistance develops through mechanisms such as secondary *EGFR* mutations, alterations to pathways downstream of or alternative to *EGFR*, or small cell lung cancer (SCLC) transformation^6^. Rarely, loss of mutant *EGFR* has been observed with TKI therapy^7–9^. Thus far, evidence for the use of immune checkpoint blockade before TKI failure is limited^10,11^, with lack of response thought to result from a lower tumour mutation burden (TMB), reduced infiltration and diminished clonal expansion of infiltrating T cells^12^. Nevertheless, new treatment approaches such as personalized chimeric antigen receptor T cell therapies^13–15^ and vaccines (for example, NCT04397926) are being explored in this patient group. There is increasing evidence that clonal neoepitopes make superior immune-based targets compared with subclonal ones^16^.

Here we present a case of loss of a clonal *EGFR* ex19del driver mutation following treatment with osimertinib and a personalized neopeptide vaccine (NPV) targeting ten somatic mutations, including *EGFR* ex19del. These targets were selected for their predicted immunogenicity, clonal nature and clinical relevance. Immuno–genomic analyses were conducted to assess immune responses to mutant *EGFR* and explore mechanisms of immune evasion and therapy resistance.

## Case report

**Fig. 1:**
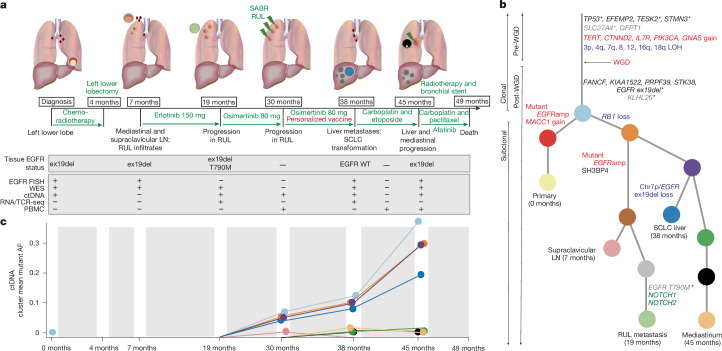
Patient pathway overview, cancer phylogenetics and ctDNA analyses. **a**, Overview of the patient pathway annotated with samples acquired and analyses performed. **b**, Phylogenetic tree of the disease. Genes in black represent the NPV-targeted mutations; grey genes represent the variants that could not be included in the vaccine owing to solubility; red represents copy number gains and blue represents losses; and genes in green are putative driver genes. The asterisks indicate the neopeptides that resulted in a GZMB response. The clonality and the timing of the mutations relative to WGD are also annotated. **c**, ctDNA mean mutant allele frequency of phylogenetic clusters. AF, allele frequency; WT, wild type; SABR, stereotactic ablative body radiotherapy; −, not available/performed; +, collected/performed; EGFRamp, EGFR amplification. **a**, Credit: J. Brock.

A 44-year-old female, a non-smoker and with no comorbidities, was diagnosed with stage IIIB poorly differentiated lung adenocarcinoma in the left lower lobe (Fig. 1a), harbouring an activating *EGFR* ex19del mutation. She underwent three cycles of neoadjuvant cisplatin/vinorelbine, followed by radiotherapy (66 Gy in 33 fractions), and a left lower lobectomy.

Three months post-operatively (month 7), imaging revealed right upper lobe (RUL) infiltrates and enlarged mediastinal and right supraclavicular lymph nodes (LNs). A supraclavicular LN biopsy confirmed disease recurrence, and erlotinib 150 mg daily was initiated. After 12 months (month 19), imaging demonstrated progression in three RUL infiltrates. Biopsy of one of the RUL lesions identified a T790M mutation, prompting a switch to osimertinib 80 mg. Although initial disease regression was observed, progression was noted in the RUL at month 30. She was treated with stereotactic ablative body radiotherapy and continued with osimertinib. She began a course of personalized neoantigen long-peptide vaccine therapy (adjuvant: montanide). Vaccine design was on the basis of whole-exome sequencing (WES) data from the supraclavicular LN (7 months) and the RUL lesion (19 months). Although 14 candidate long peptides were identified, four were insoluble in aqueous vaccine diluent (*SLC27A4*, *GFPT1*, *KLHL26* and *EGFR* T790M), and were therefore not included in vaccine production (Methods and Extended Data Table 1). Thus, four clonal pre-WGD variants (*TP53*, *EFEMP2*, *TESK2* and *STMN3*), five clonal post-WGD variants (*FANCF*, *KIAA1522*, *PRPF39*, *STK38* and *EGFR* ex19del) and one subclonal post-WGD variant (*SH3BP4*) were included in the vaccine. The *SH3BP4* variant appeared clonal at the time of manufacturing, but with different metastases subsequently sequenced, it was deemed to be subclonal (Fig. 1b).

After a further 8 months of osimertinib and five doses of intradermal vaccine, multiple liver metastases (month 38) and small mediastinal LNs emerged. Liver biopsy confirmed SCLC transformation, with no detectable *EGFR* ex19del or T790M mutations, despite 90% tumour content. Six cycles of carboplatin/etoposide achieved initial regression in the liver lesions, but within 2 months of completing treatment, disease progression in the liver and mediastinum was observed (month 45). A mediastinal biopsy revealed SCLC and the presence of *EGFR* ex19del, but not T790M. She received two cycles of carboplatin and paclitaxel but developed worsening breathlessness, requiring a bronchial stent and mediastinal radiotherapy. Pulsed afatinib (210 mg per week) was administered, but her condition deteriorated, and she died 49 months after initial diagnosis.

## Tumour evolution through treatment

**Extended Data Fig. 1:**
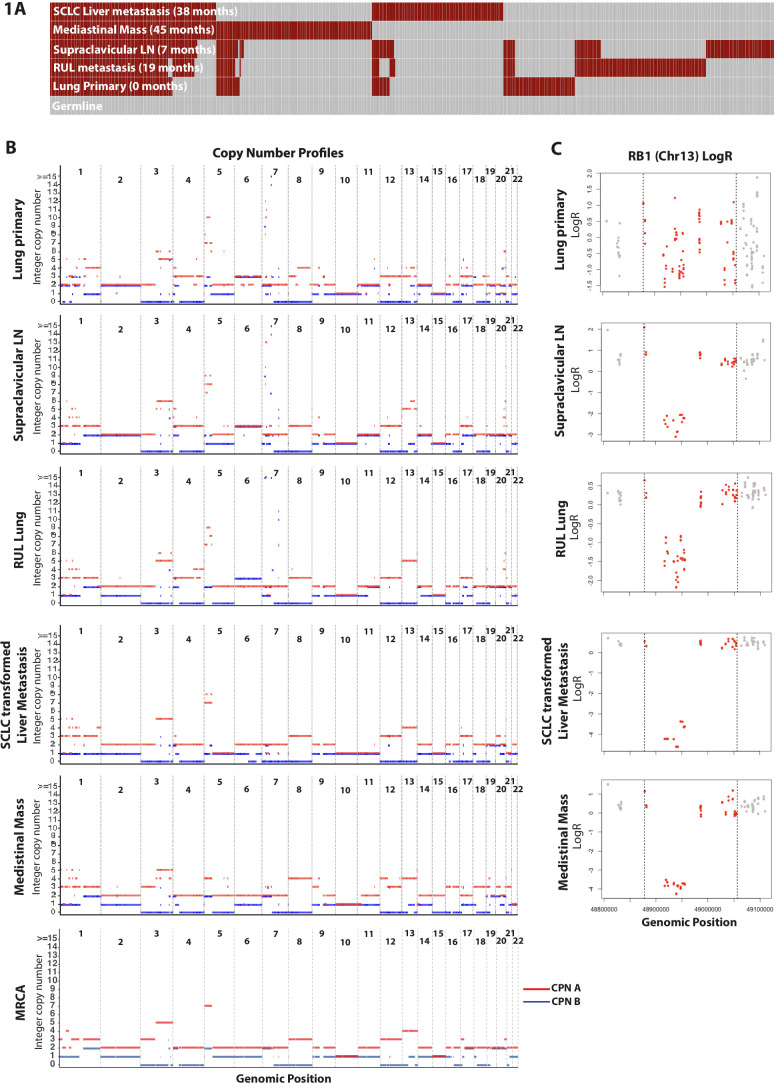
Whole exome sequencing results. **a**, Binary heatmap of mutations detected on whole exome sequencing. Each row represents a sample, and each column is a unique somatic mutation. Grey bars indicate absence of a mutation, and dark red indicates the presence of a mutation in that sample. The five samples are all clonally related as indicated by the overlap of detected somatic mutations. **b**, Phased somatic copy number aberration profiles of the whole exome sequenced samples as well as the most recent common ancestor (MRCA): diagnostic primary lung biopsy (0 months), supraclavicular LN (7 months), RUL metastasis (19 months), SCLC transformed liver metastasis (38 months), mediastinal mass (45 months) and MRCA. **c**, *RB1* LogR across all WES samples. This demonstrates evidence of deep loss of *RB1* in the supraclavicular LN, RUL metastasis, SCLC transformed liver metastasis and mediastinal mass. *RUL = right upper lobe, LN = lymph node, SCLC = small cell lung cancer*.

WES was performed on the primary lung biopsy (0 months), supraclavicular LN (7 months), RUL lesion (19 months), SCLC-transformed liver metastasis (38 months) and mediastinal mass (45 months; Fig. 1a and Supplementary Table 1). Mutation variant allele frequencies (VAFs) were integrated with local copy number and purity estimates to reconstruct the disease phylogeny (Fig. 1b and Methods). Shared clonal somatic mutations confirmed all lesions were genomically related (Extended Data Fig. 1a).

**Extended Data Fig. 2:**
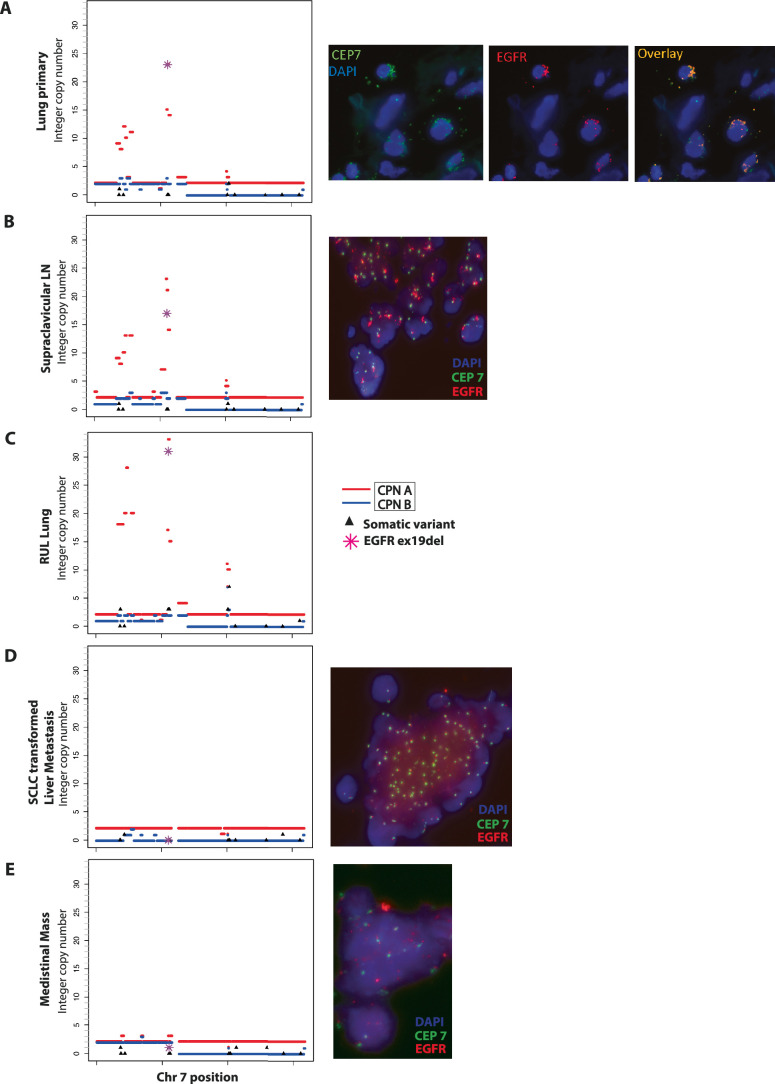
Phased somatic copy number aberration profiles of chromosome 7 encompassing *EGFR*, and associated FISH assessments. The mutant *EGFR* ex19del is represented by the magenta asterisk. Black triangles represent somatic mutations found on chromosome 7. **a**, Diagnostic primary lung biopsy (0 months). **b**, Supraclavicular LN (7 months). **c**, RUL metastasis (19 months). **d**, SCLC transformed liver metastasis (38 months). **e**, Mediastinal mass (45 months).

The somatic copy number profile of the most recent common ancestor, the cell from which all sequenced samples were descended from, was reconstructed. This revealed a clonal whole-genome doubling (WGD) event, thereby allowing the timing of mutation and copy number changes along the tumour’s phylogenetic trunk (Methods, Fig. 1b and Extended Data Fig. 1b). Several clonal mutations targeted by the vaccine (*TP53*, *EFEMP2*, *TESK2* and *STMN3*) were estimated to occur pre-WGD, as evidenced by their presence on multiple DNA copies (Fig. 1b). Similarly, loss-of-heterozygosity (LOH) events with two or more of the remaining alleles were inferred to have occurred pre-WGD (for example, LOH of 3p and 4q; Extended Data Fig. 1b). By contrast, mutations present on a single copy of doubled genomic segments occurred post-WGD; including the clonal vaccine-targeted mutations in *KIAA1522*, *FANCF*, *PRPF39* and *STK38*; and the subclonal mutation in *SH3BP4*. Interestingly, the clonal *EGFR* ex19del mutation, present at a single copy, is likely to have occurred post-WGD. Indeed, there were two copies of each of the maternal and paternal chr7p in the mediastinal mass, and only one estimated copy of the mutant ex19del allele (Extended Data Fig. 2).

Phylogenetic analyses revealed that the supraclavicular LN (7 months), RUL metastasis (19 months), SCLC-transformed liver metastasis (38 months) and the mediastinal mass (45 months) originated from a subclone with *RB1* loss identified through WES (Fig. 1b and Extended Data Fig. 1c). FoundationOne panel testing of the SCLC-transformed liver metastasis confirmed the presence of this homozygous *RB1* deletion. Homozygous *RB1* loss, coupled with *TP53* dysfunction, has been demonstrated to reduce dependence on the *EGFR* pathway for growth and is associated with SCLC transformation^17,18^. Indeed, a clonal *TP53* mutation with LOH of the wild-type allele was observed in this case. The RUL and supraclavicular LN metastases were closely related, sharing post-WGD mutations, including the subclonal vaccine-targeted mutation in *SH3BP4* (Fig. 1b). *EGFR* T790M and putative driver mutations in *NOTCH1* and *NOTCH2* were uniquely identified in a subclone exclusive to the RUL lesion that was sampled on progression to erlotinib. The liver and mediastinal masses were more closely related to each other compared with other metastases, as evidenced by shared post-WGD mutations.

Combining the phylogenetic and copy number analyses inferred two separate *EGFR* ex19del amplification events: one in the primary diagnostic biopsy, and another in the supraclavicular LN and RUL metastases (Fig. 1b and Extended Data Fig. 2a–c). Indeed, fluorescence in situ hybridization (FISH) analyses demonstrated distinct amplification patterns in these samples (Extended Data Fig. 2a,b). For the primary lung biopsy, co-occurring chromosome 7 centromere (CEP7) and *EGFR* amplification was observed, whereas in the supraclavicular LN, the ratio of *EGFR* to CEP7 probes was high. Given the co-localization of the centromere and *EGFR* gene, these amplification events are unlikely to represent extrachromosomal DNA. FISH analyses also confirmed that there was no amplification of *EGFR* in the SCLC liver metastasis or mediastinal mass (Extended Data Fig. 2d,e). Chromosomal instability, resulting in LOH of chr7p in the liver metastasis (month 38; biopsied after osimertinib therapy and vaccine therapy), is likely to have led to loss of the *EGFR* ex19del mutation (Extended Data Fig. 2d). These analyses reveal several key events in the tumour evolution, most notably the occurrence of a post-WGD ex19del in *EGFR*, different amplification events affecting the mutant *EGFR* allele, the T790M resistance mutation and loss of the *EGFR* ex19del through chromosomal instability in the *RB1-*deficient SCLC liver metastasis.

## Tracking clonal dynamics using ctDNA

Sampling the RUL lesion on progression to osimertinib at month 30 was not feasible. We therefore used circulating tumour DNA (ctDNA) as a surrogate to tissue biopsy, and investigated possible mechanisms of treatment resistance, and tracked the disease clonal dynamics.

**Extended Data Fig. 3:**
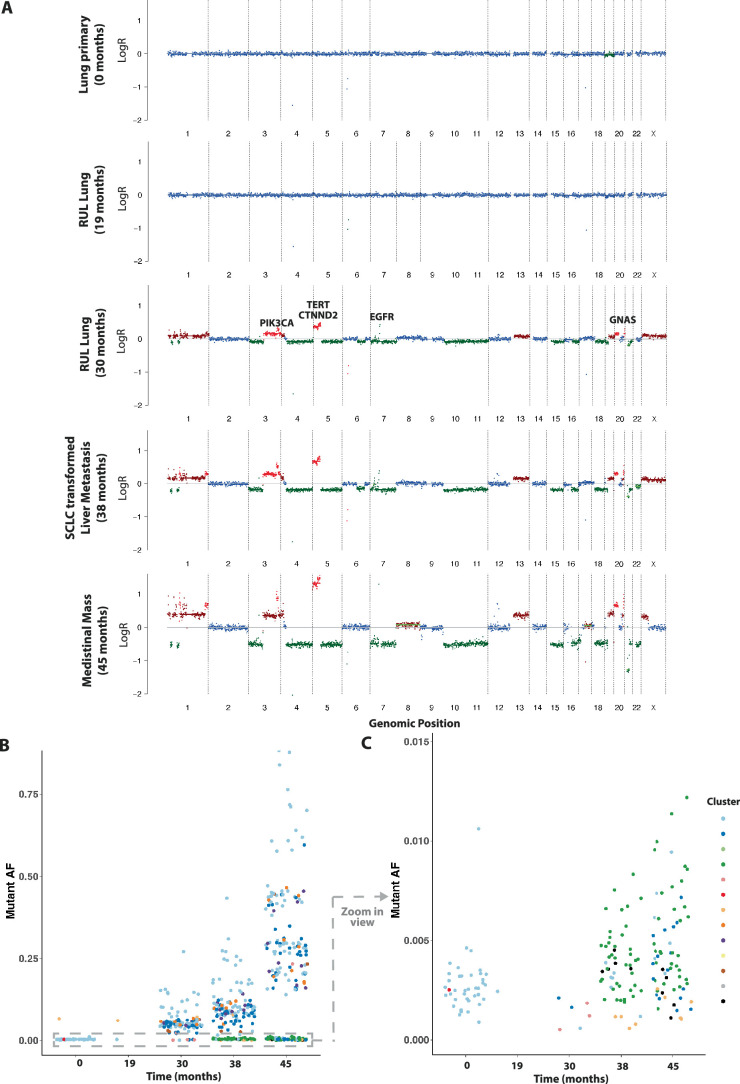
ctDNA analyses. **a**, Low pass whole genome sequencing to assess for ctDNA content and explore gains and losses. **b**, ctDNA Mutant allele frequencies (AF) at sampled timepoints. The colours reflect the phylogenetic tree clusters. **c**, Zoomed in view of the ctDNA mutant allele frequencies, focussing on AF less than 0.015. Timepoints represent primary lung biopsy (0 months), RUL metastasis (19 months), RUL progression post osimertinib (30 months), SCLC transformed liver metastasis (38 months) & Mediastinal mass (45 months) *RUL = right upper lobe, SCLC = small cell lung cancer*.

At month 30, no clear mechanism of osimertinib resistance was identified in ctDNA: alternative resistance *EGFR* mutations (for example, C797S) or driver mutations in *PIK3CA*, *KRAS*, *NRAS* and *BRAF* were not identified (Supplementary Table 2). Furthermore, no high level amplification events were detected in common resistance-associated genes (*MET*, *HER2*, *CCND1*, *CCND2*, *CCNE1*, and *CDK6* (ref. ^19^), or in *KRAS*, *NRAS*, *HRAS* or *BRAF*; Extended Data Fig. 3a). Although *PIK3CA* amplification was noted in the plasma at month 30, it preceded the development of resistance to osimertinib (Extended Data Fig. 3a and Fig. 1b). At month 30, minor subclones associated with the supraclavicular LN (pink), but not the RUL metastasis (grey and light green), were detected in the ctDNA (Fig. 1b,c). Thus, within the limit of detection for the ctDNA assay, no mechanisms of resistance could be identified^20^.

We tracked all 467 autosomal somatic variants identified in the whole-exome-sequenced lesions using ctDNA at 0, 19, 30, 38 and 45 months. (Fig. 1c and Extended Data Fig. 3b,c). At diagnosis, only the truncal clone was detectable in plasma (Fig. 1c). By month 30, at progression of the RUL lesion post osimertinib, clones from the supraclavicular LN (orange and pink; Fig. 1b,c) and the liver metastasis (orange, purple and dark blue; Fig. 1b,c) were evident in the plasma. Thus, the clone that lost the *EGFR* ex19del (dark blue; Fig. 1b,c) was identified immediately before the patient began vaccine therapy and before the radiological detection of liver metastases, implicating selection pressures imposed by osimertinib and/or pre-existing T cell immunity in the evolution of this resistant subclone. The liver-associated clones (dark blue) expanded further with continuing osimertinib, vaccine dosing and subsequent lines of therapy. Additionally, mediastinal disease-related clusters (dark green, black, pale orange) exhibited increased mean mutant allele frequencies in ctDNA at months 38 and 40.

## T cell response to vaccine therapy

**Extended Data Fig. 4:**
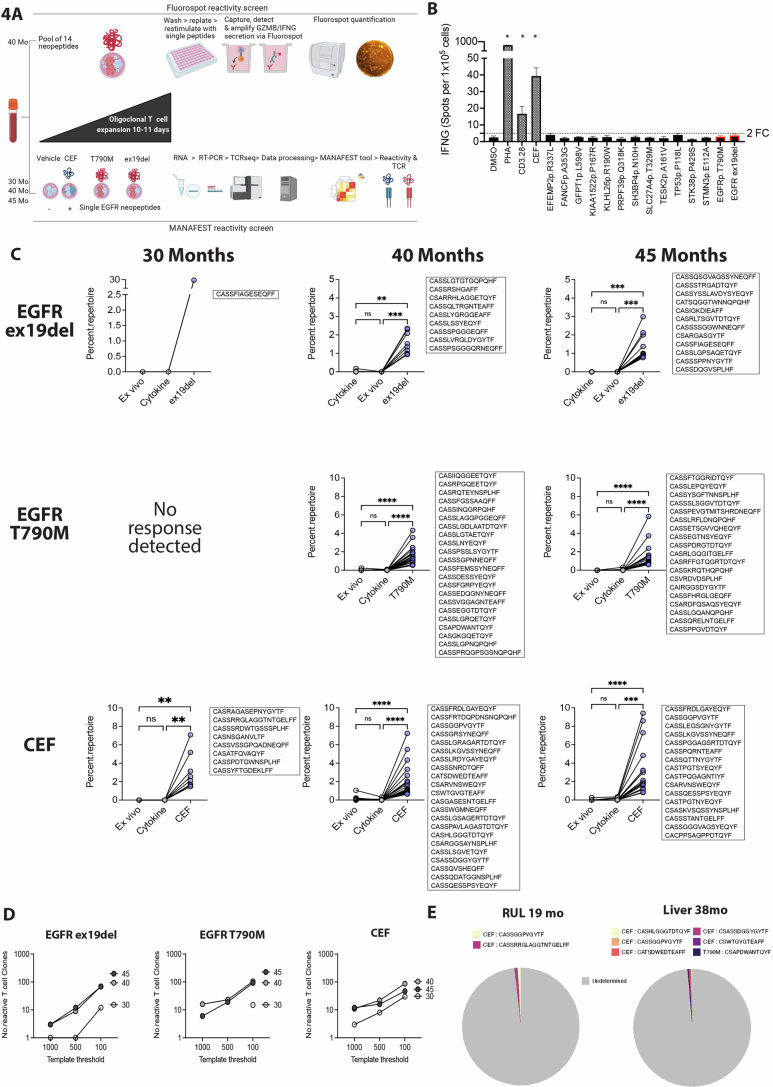
T cell reactivity analysis and TCRseq tissue profiling. **a**, Workflow for immunoreactivity assays. **b**, IFN-gamma Fluorospot at month 40; bars represent the mean +/− SEM of triplicate cultures; *P < 0.0001, one-way ANOVA. **c**, MANAFEST results showing significant clones in the conditions/time points indicated. **d**, MANAFEST parameters were adjusted over a sliding scale of expansion level for CDR3B sequences to be included in analysis (template threshold). Data show that post vaccine samples (months 40, 45) consistently harboured increased *EGFR* peptide reactivity relative to pre-vaccine timepoints (Month 30), irrespective of the template threshold used. In all analyses, clones enriched versus the cytokine alone sample and present at significantly higher levels in one condition vs all other conditions (OR > 10 and FDR < 0.01) by Fisher’s exact test, are classified as specific. **e**, TCRseq libraries of biopsies from 19 months (right upper lobe) and 38 months (liver metastasis), showing all significant CDR3s from MANAFEST assays. *CEF* = *CMV, EBV, Flu viral pools of peptides*. Illustration in **a** was created using BioRender (https://biorender.com).

To explore the immune response to the vaccine, and its potential impact on disease evolution, we analysed systemic T cell reactivity to vaccine neopeptides using granzyme B (GZMB) and interferon-γ (IFNγ) recall responses by Fluorospot. This assay exhibits enhanced sensitivity for tumour reactive T cells compared with standard ELISPOT^21,22^. Additionally, in vitro peptide stimulated clonal T cell receptor (TCR) expansion was assessed (Extended Data Fig. 4a).

**Fig. 2:**
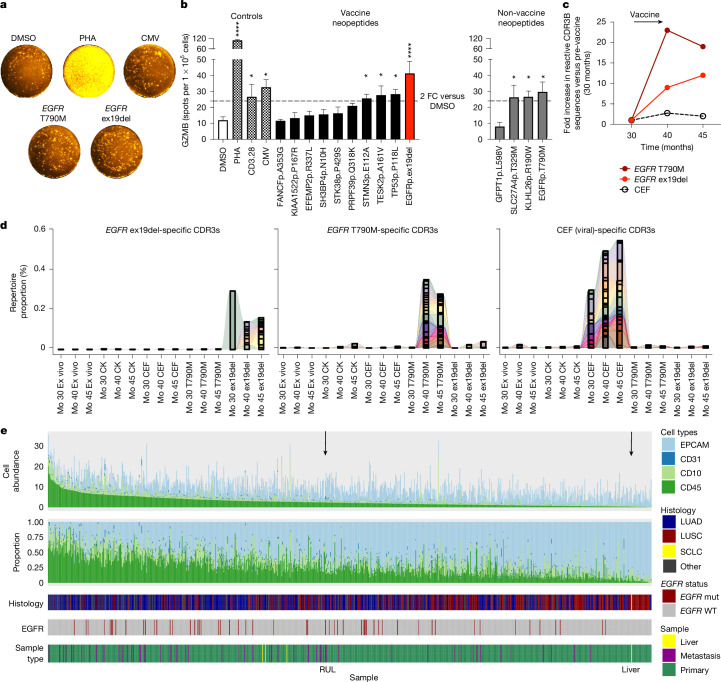
T cell reactivity to personalized neoantigen vaccine epitopes. **a**, Representative images from GZMB Fluorospot recall assay testing vaccine and non-vaccine neopeptides in PBMCs at 40 months (post-vaccine). **b**, Quantification of GZMB release by Fluorospot (40 months); bars represent the mean ± s.e.m. of triplicate cultures; *pAdj < 0.05, ****pAdj < 0.0001, one-way analysis of variance corrected for multiple testing by Benjamini–Hochberg. **c**, Fold change in the number of specific CDR3 beta chain sequences detected for *EGFR* and viral (CEF) peptides by MANAFEST in PBMCs sampled over time points shown (months, *x* axis), calculated relative to pre-vaccine (month 30); fold change set to 1 for no detectable T790M clones at month 30. **d**, The proportion of the repertoire in each sample that is occupied by specific CDR3s (shown as bar segments) reactive to peptides indicated at various time points as determined by MANAFEST. Connecting waves indicate clonotype sharing between samples. **e**, CIBERSORTx scores for the TRACERx 421 cohort and both RUL and SCLC-transformed liver metastasis (TRACERx 421, number of tumour regions, *n* = 954 regions from 347 patients), exploring the abundance of stromal (CD10^+^ and CD31^+^), immune (CD45^+^) and epithelial/cancer cells (EPCAM^+^). PHA, phytohaemagglutinin; FC, fold change; CK, cytokine; Mo, month; LUAD, lung adenocarcinoma; LUSC, lung squamous cell carcinoma.

Initially, Fluorospot was used to assay peripheral blood mononuclear cells (PBMCs) collected 2 months after vaccination (month 40). Significant GZMB responses were observed for four of ten neopeptides contained in the vaccine (clonal pre-WGD: *TP53*, *TESK2*, *STMN3*; clonal post-WGD: *EGFR* ex19del; adjusted *P* value (pAdj) < 0.05 versus dimethylsulfoxide (DMSO); Fig. 2a,b). Among these, *EGFR* ex19del elicited the strongest response (41 specific spots, pAdj < 0.0001), which was comparable to positive controls (anti-CD3, anti-CD28, 26 specific spots; cytomegalovirus (CMV) peptides, 32 specific spots; Fig. 2b). No GZMB responses were detected for the other six epitopes, which may be due to suboptimal neoantigen prediction, low immunogenicity and/or tissue sequestration/migration of cognate T cells. There was no association between GZMB response and neoepitope clonality (Fisher’s exact test, 4 of 9 clonal with GZMB response versus 0 of 1 subclonal; *P* = 1), or genome doubling status of the vaccine-targeted mutations (Fisher’s exact test, 3 of 4 pre-WGD with GZMB response versus 1 of 6 post-WGD; *P* = 0.19). To explore the possibility that the patient may have developed T cell responses to additional, non-vaccine neopeptides, we assayed the four neoantigens predicted to be immunogenic from WES data that were not included in the final vaccine. Three of these peptides (derived from mutant *SLC27A4*, *KLH26* and *EGFR* T790M) elicited a significant GZMB Fluorospot response (pAdj < 0.05; Fig.2b), suggesting that the patient had mounted additional neoantigen-specific T cell responses spontaneously or as a result of epitope spreading. No IFNγ secretion was detected in response to any epitope (Extended Data Fig. 4b), consistent with the enhanced sensitivity of GZMB for detecting tumour-reactive T cells^21,22^ whilst also potentially reflecting impaired T cell polyfunctionality and/or suboptimal neopeptide priming or recall.

Given that the *EGFR* ex19del specific GZMB response was detected post vaccination, we sought to determine whether this reactivity was vaccine elicited. We therefore tracked the maturation of the *EGFR* ex19del-directed neoantigen-specific T cell response in PBMCs collected pre- (month 30) and post-vaccine (months 40 and 45). To do so, we used the mutation-associated neoantigen functional expansion of specific T cells (MANAFEST) assay^23^, a sensitive method which allows serial tracking of antigen-specific responses through unique TCR CDR3B sequences that clonally expand after in vitro culture with neopeptides (Extended Data Fig. 4a).

**Extended Data Fig. 5:**
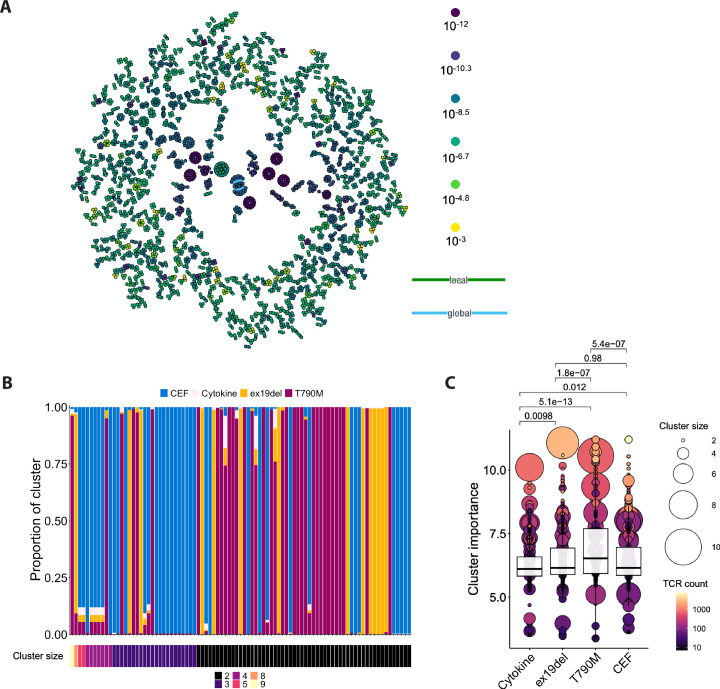
TCRseq clustering of *EGFR* neopeptide stimulated MANAFEST cultures. To identify groups of TCRs that shared similar sequence structure to the clones that were significantly expanded in PBMC samples from the MANAFEST assay, we clustered together the top 3000 CDR3B sequences from each timepoint (months 30, 40, 45) from each condition (Cytokine, CEF, ex19del, T790M), using the Gliph2 clustering algorithm. **a**, Gliph2 network plot, coloured by ‘total.score’ metric, herein referred to as cluster importance score. More significant clusters are located towards the middle of the plot. **b**, Overview of clusters which contain expanded sequences, as defined by the MANA webtool. As all samples were clustered together, the proportion of each condition’s contribution to the cluster is displayed in the bar. Cluster size refers to the number of unique TCR sequences within each cluster. We observed that clusters containing an expanded sequence determined by the MANA webtool were composed of sequences predominantly of the same condition. **c**, Clusters were segregated into conditions using a proportion cut-off of 50%. All clusters from Gliph2 output which had a sequence count above the threshold are displayed. Significance determined by two-tailed Wilcoxon test. There was no difference in cluster importance scores between the ex19del culture and the positive control (CEF), whilst the T790M condition showed the highest cluster importance score. Taken together, these data suggest that the expanded clones in their respective cultures were driven by peptide-specific stimulation, as we see more significant clustering in the conditions treated with peptide compared to cytokine alone. The box plots represent the upper and lower quartiles (box limits), the median (centre line) and the vertical bars span the 5th to 95th percentiles.

Pre-vaccination (30 months), we detected reactivity to a cocktail of common major histocompatibility complex class I restricted CMV, Epstein–Barr virus (EBV) and influenza viral recall peptides (CEF) used as a positive control (eight expanded TCR sequences; Extended Data Fig. 4c). The number of bystander CEF reactive TCRs exhibited a modest 2–2.75-fold increase through vaccination at months 40 and 45 (22 and 16 significantly expanded clones, respectively; Fig. 2c,d). The *EGFR* ex19del neopeptide induced a significant expansion of a single TCR sequence in pre-vaccination PBMCs (Fig. 2d and Extended Data Fig. 4c, bottom row). Post-vaccination we observed 9 and 12 significantly expanded ex19del-specific TCRs at 40 and 45 months, respectively; Fig. 2c,d). Interrogating TCR clonal dynamics revealed that the pre-vaccination *EGFR* ex19del TCR clonotype persisted in circulation for over a year through therapy (Fig. 2d and Supplementary Data). Similarly, viral-specific memory T cell clones were maintained long term (for example, five significantly expanded TCRs in response to CEF in more than one time point, three of which were preferentially amplified through CEF stimulation across all time points; Fig. 2d and Supplementary Data). For *EGFR* T790M, a neopeptide not included in the vaccine but reactive in Fluorospot at month 40, no significant TCR reactivity was identified pre-vaccine at month 30, despite the presence of the mutation in the RUL at month 19. However, significantly expanded T790M-specific TCRs were detected at months 40 and 45 (22 and 19 significantly expanded TCRs, respectively), including two CDR3Bs maintained across both post-vaccination time points (Fig. 2c,d and Supplementary Data). An increase in post vs pre-vaccine reactive TCRs was maintained across a sliding scale of MANAFEST assay thresholds (Extended Data Fig. 4d). Additionally, significantly expanded TCRs identified by MANAFEST exhibited peptide-specific TCR amino acid sequence convergence, recently shown to indicate common antigen specificity in non-small cell lung cancer (NSCLC)^24^ (Extended Data Fig. 5a–c).

Taken together, the MANAFEST and Fluorospot results indicate T cell reactivity to both the *EGFR* ex19del and the T790M neopeptides. The responses suggest generation of new clones (ex19del) and/or induction (T790M) of reactivity at 40–45 months, coinciding with vaccination, radiotherapy and continuing osimertinib therapy. Although the new ex19del-specific TCRs present at months 40–45 may be the result of vaccination, the T790M-reactive TCRs emerging at this time likely reflect epitope spreading, which can arise from both neoantigen vaccination^25^ and radiotherapy^26^.

## Loss of targeted neoantigens at resistance

**Extended Data Fig. 6:**
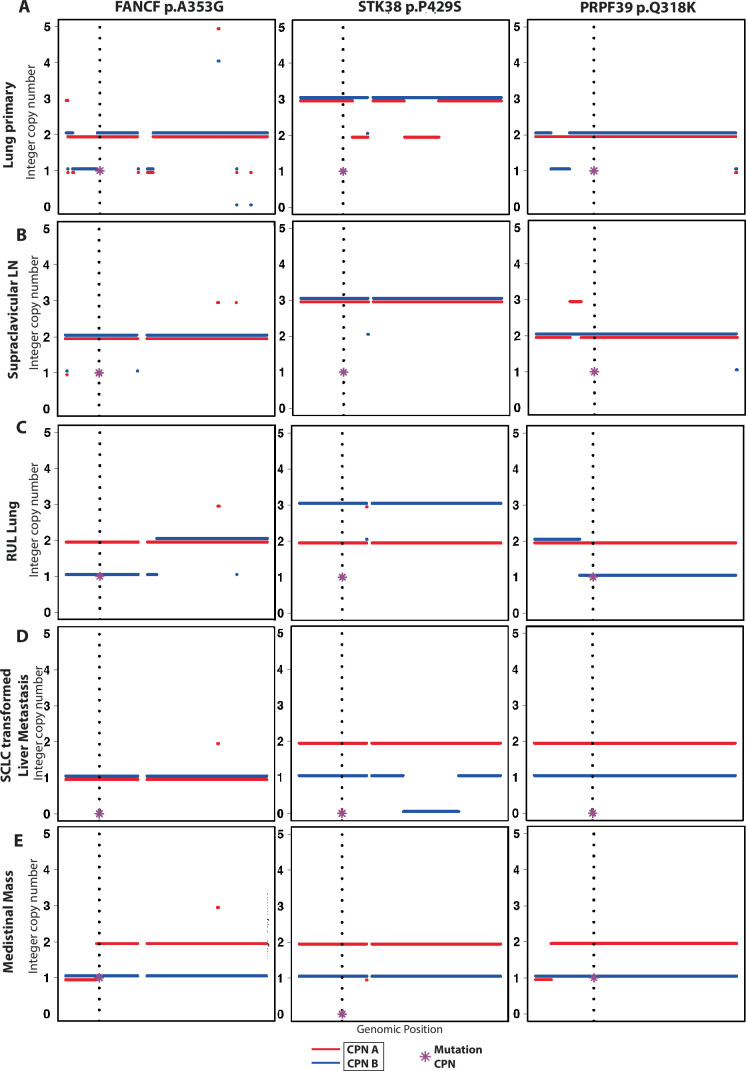
Copy number profiles for post-WGD lost variants. Copy number changes observed at the genomic positions for the post-genome doubling variants *FANCF* p.A353G, *STK38* p.P429S and *PRPF39* p.Q318K in the: **a**, lung primary; **b**, supraclavicular LN; **c**, RUL lung; **d**, SCLC transformed liver metastasis; **e**, mediastinal mass. The dotted lines represent the genomic positions of the mutations.* RUL = right upper lobe, SCLC = small cell lung cancer*.

Five of the ten vaccine-targeted mutations were absent in the resistant liver metastasis that emerged after radiotherapy, osimertinib and vaccination (month 38; missing targets: *FANCF*, *STK38*, *SH3BP4*, *PRPF39*, *EGFR* ex19del). Strikingly, these mutations all occurred post-WGD, whereas the four pre-WGD vaccine-targeted mutations remained present (5 of 6 post-WGD lost versus 0 of 4 pre-WGD; Fisher’s exact test, *P* = 0.0476; Extended Data Table 1). Further exploration of the mechanism of loss revealed that *SH3BP4* was a subclonal mutation, found in a separate branch seeding the RUL and supraclavicular metastases (Fig. 1b). For the clonal post-WGD variants (*FANCF*, *PRPF39*, *STK38* and *EGFR* ex19del), loss was due to copy number alterations (Extended Data Figs. 2d and 6). These results suggest that post-WGD mutations are more vulnerable to loss through chromosomal instability or tumour heterogeneity, making pre-WGD mutations more reliable targets for future therapies.

## Exploring mechanisms of immune failure

Despite the GZMB reactivity against multiple neoepitopes, the patient’s disease progressed, suggesting underlying mechanisms of immune failure.

**Extended Data Fig. 7:**
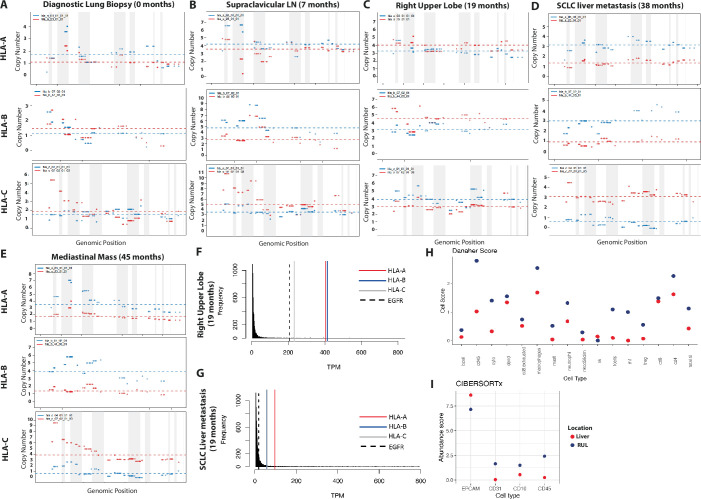
Bioinformatics immune analyses. HLA copy number analyses for the various tissue-sequenced timepoints. There is no evidence of loss of heterozygosity in *HLA-* *A*, *B* or *C* for the: **a**, diagnostic primary lung biopsy (0 months); **b**, supraclavicular LN (7 months); **c**, RUL metastasis (19 months); **d**, SCLC transformed liver metastasis (38 months); **e**, mediastinal mass (45 months). TPM distribution for all expressed genes from RNA at the sequenced time points at: **f**, 19 months (RUL metastasis) **g**, 38 months (SCLC liver metastasis). Expression of *EGFR* and *HLA-A, B & C* are highlighted in black, red, blue and grey respectively. **h**, Danaher signature scores. Results demonstrate a reduction in CD45+ cells as well as many other immune cell types in the SCLC liver-transformed metastasis (post osimertinib and vaccine; 38 months) compared to the RUL metastasis (post erlotinib; 19 months). **i**, CIBERSORTx cell abundance scores. Similar to the Danaher scores, these results support that CD45+ cells (immune cells) are less abundant in the SCLC transformed liver metastasis compared to the RUL metastasis. TPM, transcripts per million.

No evidence of human leukocyte antigen (HLA) LOH^27^, or mutations affecting HLA peptide-processing, interferon-signalling or checkpoint inhibition pathways, was identified at any sequenced time point (Extended Data Fig. 7a–e and Supplementary Tables 3 and 4). Bulk RNA sequencing (RNA-seq) from the RUL metastasis (19 months; 49% tumour content) and SCLC liver metastasis (38 months; 91% tumour content) demonstrated no global *HLA-A, -B* or *-C* transcript repression (Extended Data Fig. 7f,g). However, bulk RNA may reflect both cancer and microenvironment cell expression. Despite the *TP53*, *TESK2* and *STMN3* neopeptides (included in the NPV) eliciting a GZMB response, the corresponding DNA mutations remained detectable in the liver metastasis and therefore we explored the RNA-seq data for evidence of transcript repression. Mutant *TP53* and *TESK2* were identified in the liver RNA-seq data (RNA VAF: 95.2% and 73.3%, respectively). However, the exon containing the *STMN3* variant had very low coverage, despite other exons in the gene showing higher coverage, and we therefore cannot rule out transcript repression.

Immune infiltration analyses revealed that histological tumour infiltrating lymphocyte (TIL) scores^28^ were lower in the liver metastasis (5%) compared with the RUL metastasis (20%). This reduction was confirmed by Danaher gene signature^29^ results (total TIL score: liver 0.42 versus RUL 1.12) and CIBERSORTx^30^ (CD45^+^ abundance score: liver 0.25 versus RUL 2.42; Extended Data Fig. 7h,i). Additionally, *CXCL9* expression, critical for T cell recruitment and a biomarker for checkpoint inhibitor response^31^, was over tenfold lower in the liver metastasis (transcript per millions (TPM): liver 0.38 versus RUL 8.73; Supplementary Table 5).

TCR repertoire analysis of the liver metastasis did not recover *EGFR* ex19del-targeting TCRs previously identified by the MANAFEST assay, although a single *EGFR* T790M reactive clone was detected. Four MANAFEST-defined viral reactive T cell clones were found in both RUL tissue (19 months) and the liver metastasis (38 months), suggesting bystander infiltration (Extended Data Fig. 4e), but a paucity of neoantigen-reactive T cells. However, false negatives cannot be ruled out because of stochastic sampling in limited tissue biopsies.

These findings demonstrate limited inflammatory cell recruitment, a known mechanism of immune evasion in liver metastasis, as shown in mouse models and immunotherapy-treated patients with NSCLC^32^. Taken together, the observed post-WGD neoantigen loss and the hostile microenvironment are factors that may have impaired neoantigen surveillance despite systemic neoantigen reactivity.

## Microenvironment comparison with TRACERx 421

Liver metastases are known to exhibit poor immune infiltration^32^. To provide context, we compared the TIL scores and RNA-seq results from this case report with the TRACERx 421 cohort, a longitudinal study of early-stage NSCLC with multiregion primary and relapse tissue sampling^3,33^. The cohort included 432 primary tumours (1,554 WES regions)^3^, with representative primary tumour TIL scores available for 409 of these tumours; and RNA-seq data from 954 tumour regions across 347 patients (893 primary regions, 29 LNs, 2 satellite lesions, 30 recurrence/progression samples)^34^. There were 28 cases harbouring *EGFR* mutations (11 ex19dels, 7 L858R, 4 exon 20 insertions, 2 L861Q, 1 V834L, 1 G719C, 1 G719A, 1 exon 17 deletion).

**Extended Data Fig. 8:**
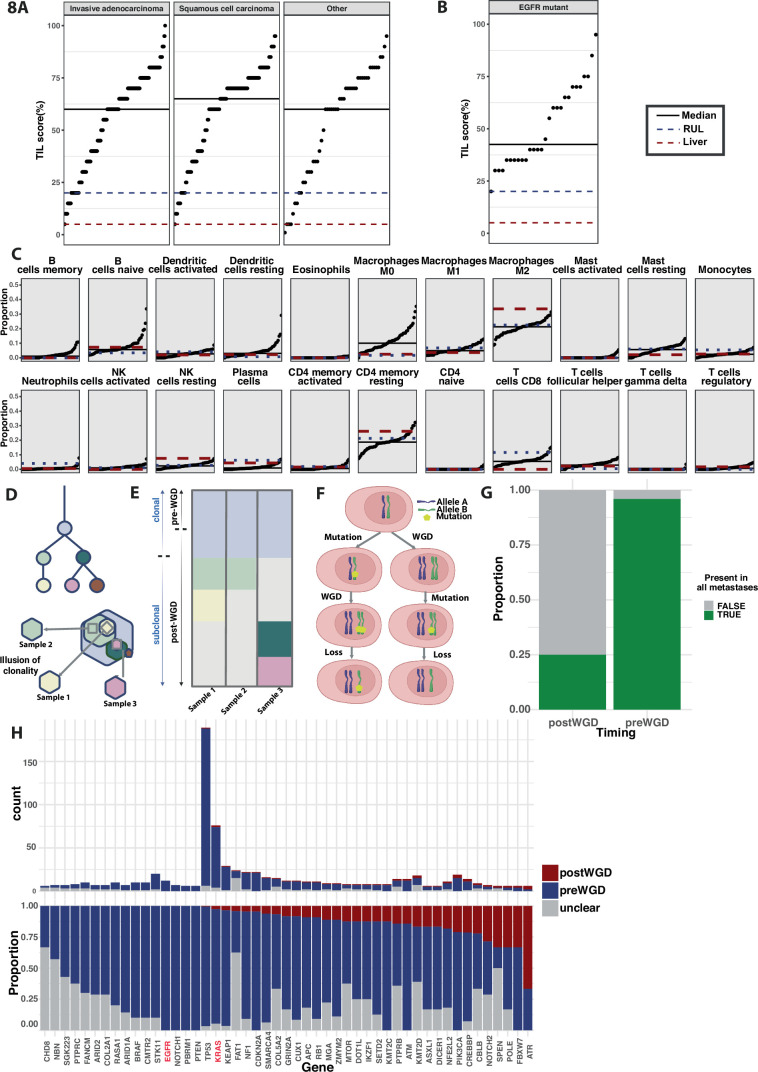
TRACERx 421 immune and genomic data. **a**, Comparison of histological tumour infiltrating lymphocyte (TIL) scores of the RUL and liver metastasis with primary tumours from the TRACERx 421 cohort (invasive adenocarcinoma n = 232, Squamous cell carcinoma n = 131, Other histology n = 46). **b**, Comparison of TIL scores with *EGFR*-mutant NSCLC cases within the TRACERx 421 cohort (n = 28). **c**, Using CIBERSORTx, the proportion of immune cells in all regions of the *EGFR*-mutant TRACERx 421 cohort with RNASeq data was calculated using the LM22 signature. The right upper lobe (RUL) and liver metastasis from this case are represented with dotted lines (blue and red, respectively). A solid black line represents the median of the *EGFR*-mutant TRACERx 421 tumours (n = 23 EGFR-mutant cases, with 54 primary tumour samples, 4 of which are metastatic). **d**, Illusion of clonality from single region biopsies; variants can appear to be clonal when they are in fact subclonal at the tumour level. **e**, Mutation heatmap for biopsies shown in panel. Whole-genome doubling (WGD) is usually an early clonal event in NSCLC, therefore, pre-WGD are likely to also be clonal. **f**, Mutations occurring post-WGD can be lost more easily than pre-WGD mutations through chromosomal instability as they occur on 1 chromosome. **g**, Mutations occurring pre-WGD, were more likely to be found in every metastasis sequenced than post-WGD mutations (96.7% versus 25.3%). **h**, Summary of clonal lung cancer driver gene SNV or DNV mutations timed relative to WGD. This only has genes with at least five mutations in the TRACERx 421 cohort. Top panel represents the number of mutations; lower panel represents the proportions of the variants in that gene. Dark red represents post-WGD, grey represents unclear timing & dark blue represents pre-WGD. *WGD = whole genome doubling; RUL = Right upper lobe.* Illustrations in **d**–**f** were created using BioRender (https://biorender.com).

The RUL metastasis (20%) and SCLC-transformed liver metastasis (5%) had low TIL scores, corresponding to the 10th and <5th centiles of the TRACERx 421 distribution (median TIL score = 60%, interquartile range (IQR) = 35–75%; Extended Data Fig. 8a,b). Notably, the SCLC liver metastasis had the lowest TIL score compared with any of the 28 *EGFR* TRACERx mutant cases scored (median 42.5%, IQR = 35–66.25%, minimum = 20%; Extended Data Fig. 8b).

To validate these findings, we implemented CIBERSORTx^30^ to analyse multiregion RNA-seq data from the TRACERx 421 cohort, assessing immune (CD45^+^), epithelial/cancer (EpCAM^+^) and stromal (CD10^+^ or CD31^+^) cell abundance. The median CD45^+^ cell abundance score in the TRACERx cohort was 2.14 (IQR = 1.05–3.77; Fig. 2e). The SCLC liver metastasis had a very low score (0.253) compared with the whole cohort (<5th percentile) and three liver metastatic regions from two TRACERx patients (2.884, 2.696 and 2.897). It also showed lower immune infiltration compared with 54 RNA-seq-analysed *EGFR* mutant regions (*n* = 23 cases; median CD45^+^ abundance 2.65; IQR = 1.96–4.44; minimum = 0.4339).

We next used the LM22 signature matrix^35^, a validated gene signature matrix of 22 haematopoietic cell types, to compare the immune composition of the SCLC-transformed liver with the *EGFR* mutant samples in the TRACERx cohort. The SCLC liver metastasis exhibited a lower proportion of CD8^+^ T cells (SCLC liver CD8^+^ proportion 0; TRACERx *EGFR* mutant cohort median = 0.054, IQR = 0.037–0.071) and higher proportions of immunosuppressive M2 macrophages (SCLC liver M2 macrophages proportion 0.34; TRACERx *EGFR* mutant cohort median = 0.21, IQR = 0.18–0.24; Extended Data Fig. 8c). Thus, compared with the RUL lesion and TRACERx *EGFR* mutant samples, the SCLC liver metastasis displayed both lower immune infiltration and a more hostile microenvironment, characterized by fewer CD8^+^ T cells and increased M2 macrophages.

## Improving target selection from single biopsies

Including clonal antigens in vaccine or adoptive T cell therapy design is thought to enhance neoantigen targeting^16^. However, identifying true clonality from single biopsies performed in clinical practice is challenging because of regional selective sweeps, which can create an ‘illusion of clonality’ in which a mutation seems clonal locally but is subclonal overall^36,37^ (Extended Data Fig. 8d). Because WGD is typically an early clonal event in NSCLC^38^, pre-WGD mutations are more likely to be truly clonal, whereas post-WGD mutations may be clonal or subclonal. Furthermore, as observed in this case report, pre-WGD mutations are conceivably less prone to loss through chromosomal instability owing to their presence on multiple chromosome copies (Extended Data Fig. 8e,f). Indeed, persistence of variants has been shown to be important for effective neoantigen targets^39^. Therefore, using the TRACERx 421 cohort, we assessed whether mutations defined as pre-WGD from a single biopsy are enriched for true clonal status, and whether they are less prone to loss during tumour evolution^3,33^.

**Fig. 3:**
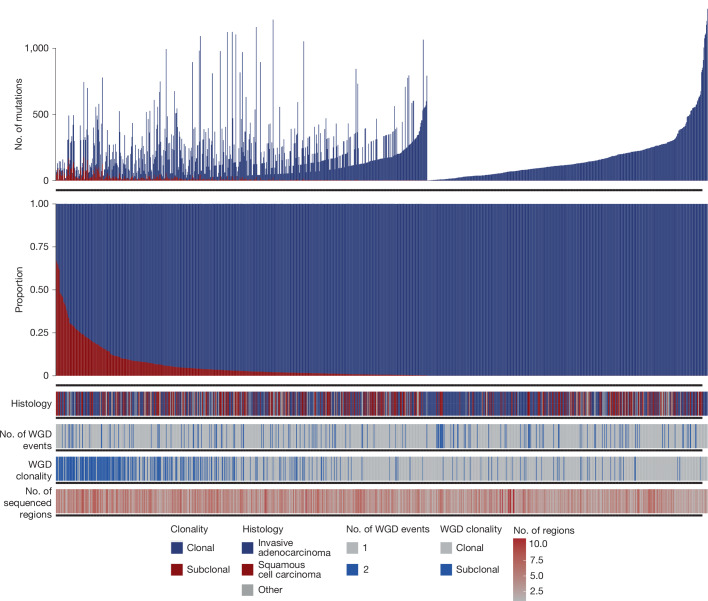
TRACERx 421 cohort analyses. Clonality of all pre-WGD mutations from tumour regions with evidence of WGD. Each column represents a single region from the TRACERx 421 cohort. The median proportion of pre-WGD mutations that were also clonal at a region level is 99.4% (IQR = 96.6–1). Regions in which pre-WGD mutations have a high proportion of subclonal mutations are enriched for tumours with subclonal WGD events and tumours that have a higher number of sequenced regions.

To mimic clinical sampling, each tumour region was treated as an independent biopsy. Variant timing relative to WGD was calculated (Methods), and compared with the variant clonality as defined by whole-tumour phylogenetics^3^. Of the 432 tumours sequenced in the TRACERx 421 cohort, 403 tumours (1,428 regions) had at least 2 regions with sufficient purity to perform copy number analyses. Of these, 1,085 regions (from 307 tumours) had at least one estimated genome doubling event (933 regions with one WGD event and 152 regions with two events). Using these 1,085 regions, we analysed 750,216 single or dinucleotide variants: 229,325 pre-WGD, 192,043 post-WGD and 328,848 variants with unclear timing. Most of the pre-WGD variants (221,096/229,325; 96.4%) were found to be clonal, compared with only 31.4% of post-WGD variants (60,282/192,043), confirming that pre-WGD variants are predominantly clonal in NSCLC (Fisher’s exact test, *P* = 0; Fig. 3).

Regions with a low proportion of clonal pre-WGD mutations were associated with subclonal WGD events (Fig. 3; Supplementary Note). Among 307 tumours with WGD, 78 (19%) had evidence of a subclonal WGD. Even in these cases, the median proportion of pre-WGD mutations also classified as clonal remained high at 94.2% (IQR = 83.5–98.8%), reinforcing pre-WGD mutations as reliable markers of clonality. Previously, we showed that pre-WGD mutations are more likely to persist in metastases compared with post-WGD or non-WGD primary-tumour-ubiquitous mutations^31^, making them strong candidates for immune-based therapies. Indeed, for primary tumours with WGD events, we found that only 25.3% (11,412 of 45,189) of post-WGD variants were identified in all sequenced metastases, versus 96.7% (28,140 of 29,108) of pre-WGD variants (Fisher’s exact test, *P* = 0; Extended Data Fig. 8g).

## Timing of clonal driver events

In this case, the *EGFR* ex19del, typically associated with early lung cancer development, was estimated to have occurred post-WGD, highlighting its potential vulnerability to loss as a resistance mechanism. To assess how often *EGFR* driver events occur post-WGD, we explored the TRACERx 421 dataset.

Among 28 *EGFR* mutant NSCLC cases in the TRACERx 421 cohort, 20 showed evidence of genome doubling (19 clonal and 1 subclonal first WGD events; with 3 clonal and 2 subclonal second WGD events). All *EGFR* variants were deemed to be clonal. No strong evidence of post-WGD *EGFR* activating variants was found, suggesting that such events are uncommon (Extended Data Fig. 8h).

Expanding the analysis to all clonal driver mutations, we assessed 1,119 single- and dinucleotide variants from 189 genes across 291 tumours (first WGD event was clonal in 246 and subclonal in 45 cases). Of these, 84 (7.5%) occurred post-WGD and 854 (76.3%) pre-WGD; 181(16.1%) could not be clearly timed (Extended Data Fig. 8h). Notably, two clonal KRAS mutations (Q61R and G12S) occurred post-WGD, both in cases with two WGD events, and appearing after the first WGD. As targeting driver mutations in TKIs, vaccines or adoptive cell therapies^40,41^ gains traction, understanding the timing of mutations is critical.

## Discussion

The proportion of lung cancer in never smokers is increasing^42^ and is often linked to drivers in genes such as *EGFR* and *ALK*. Optimizing outcomes requires an improved understanding of resistance mechanisms to TKIs, vaccinations and immune therapies. Using genomics and functional immune responses, we report the loss of an *EGFR* ex19del in a patient treated with both osimertinib and a personalized peptide vaccine targeting multiple mutations, including *EGFR* ex19del. Whereas loss of T790M after treatment with osimertinib is a known mechanism of resistance^6^, loss of the initial *EGFR* driver mutation (ex19del or L858R) has been reported only in isolated cases^7–9^ and cell lines^43^.

The low mutation burden usually seen in *EGFR* mutant lung cancers is thought to limit antigenicity and immune surveillance, with regulatory T cell-mediated suppression^44^ or immune exclusion potentially impeding T cell responses further. However, we identified an *EGFR* ex19del directed T cell response via GZMB Fluorospot and MANAFEST assays, including a MANAFEST-defined *EGFR* ex19del reactive TCR present in the patient’s circulation before vaccination, which persisted for more than a year. High-resolution ctDNA phylogenetic analyses revealed that the cancer subclone which had lost the *EGFR* ex19del variant emerged before vaccination and continued to expand during osimertinib and vaccine exposure. This pre-existing immune response may have contributed to the selection of a minor *EGFR* wild-type subclone, which may have gained a growth advantage during osimertinib and/or vaccine therapy, especially in the context of homozygous *RB1* deletion reducing dependence on the *EGFR* pathway^17,18^. Consistent with this reponse, previous reports have also demonstrated that *EGFR* ex19del neoantigens can evoke T cell responses in patients receiving immunotherapy^44,45^.

 We observed GZMB Fluorospot responses in 7 out of 14 neoepitopes assayed, highlighting circulating reactivities that may have emerged spontaneously, or as a direct or indirect result of vaccination. MANAFEST data suggest that the NPV may have primed new ex19del-specific TCR clones, while inducing T790M-specific TCRs via epitope spreading. We note that at lower stringency MANAFEST analysis calls TCRs reactive to both epitopes pre-vaccination. However, the relative increase in reactive clones post vaccination remained consistent supporting the conclusion that the NPV enhanced and/or induced reactivities to ex19del and T790M most probably directly or indirectly, respectively. However, we cannot exclude the possibility that radiotherapy or TKIs played a role in the induction or diversification of these T cell responses. Furthermore, limited blood volumes at the time of analysis prevented us from exploring whether wild-type sequences in the long peptides contributed to reactivity, or whether the responses were derived from CD4^+^ or CD8^+^ T cells. Ultimately, the NPV failed to prevent disease progression, underscoring increasingly recognized limitations of using systemic T cell reactivity as a surrogate for vaccine efficacy. In this instance the absence of IFNγ in the presence of a GZMB response in Fluorospot analysis suggests that neoantigen-specific T cells may have been sub-optimally primed and/or recalled.

The immune desert phenotype, often seen in liver metastases, can suppress immunotherapy responses^32^. We observed low overall immune cell infiltration and *CXCL9* expression, a key chemokine for recruiting CXCR3^+^ T cells and a strong biomarker of checkpoint inhibitor response^31^. Additionally, there was a high proportion of immunosuppressive M2 macrophages in the progressing SCLC-transformed liver metastasis. Notably, systemic neoantigen reactive T cells can be trafficked into liver metastases and apoptosed by FASL-expressing intra-hepatic M2-like macrophages^32^.

In this case, post-WGD vaccine-targeted variants were more frequently lost in the metastatic SCLC liver metastasis than pre-WGD mutations through chromosomal instability and/or selection of subclones from different branches of the phylogenetic tree. Genome doubling is often an early/truncal event in NSCLC^38^. Conceivably, it is easier to lose a post-WGD mutation located on one chromosome copy than pre-WGD mutations which are probably found on multiple copies of the chromosome. This may make pre-WGD variants better targets for personalized immune therapies. Although case reports have limited generalizability, analysis of the TRACERx 421 dataset confirms that pre-WGD mutations more likely to represent clonal variants and are less likely to be lost in metastases than post-WGD variants. Thus, targeting pre-WGD mutations to avoid neoantigen loss through TKI or immune selection pressure, combined with strategies to enhance immune cell infiltration, may improve outcomes. Promising neoantigen vaccine responses, particularly with checkpoint blockade, suggest that therapeutic tumour vaccines may require combination immunotherapies^46^. Overall, these data highlight the value of phylogenetic disease tracking and T cell profiling to understand immune escape and therapy failure.

## Methods

### Study oversight

Clinical oversight of the vaccine therapy was undertaken at University Medical Centre, Heidelberg. The patient was treated with a personalized peptide vaccine within the scope of an individual healing attempt (statement WD 9–3000–083/23 of the German Parliament, guidelines 2001/20/EG and 2005/28/EG, Declaration of Helsinki of the World Medical Association (Article 37)); approval by the Institutional Review Board and ethics committees is not required. Informed consent for the vaccine therapy was taken in accordance with local policies. Informed consent for genetic and immune research studies was obtained in accordance with protocols approved by the University Medical Centre Heidelberg Institutional Review Board. Written, informed consent to transfer and perform analyses at the Francis Crick Institute and associated institutions was also provided.

### DNA sample extraction and sequencing

#### Fresh frozen and FFPE samples

The methods for DNA extraction and sequencing for fresh frozen and formalin-fixed paraffin-embedded (FFPE) samples are summarized in the TRACERx manuscripts^3,33,38^. For fresh frozen recurrence/progression samples, paired germline DNA was re-sequenced in the same run, using germline DNA from aliquots extracted at initial germline blood collection. No further germline sequencing was performed for FFPE samples.

### WES bioinformatics pipeline

The bioinformatics pipeline, including quality control checks, used for WES data analysis is summarized in the TRACERx manuscripts^3,33,38^. VarDict (v.2016.11.21) was used to call the VAF of the *EGFR* ex19del, as it has been shown to have improved estimates of indel allele frequencies^47^.

### Phylogenetic trees

CONIPHER (COrrecting Noise In PHylogenetic Evaluation and Reconstruction) was used to construct the phylogenetic tree^3,48^. The tree was manually reviewed/selected, and orthogonal checks were performed (Supplementary Note).

### Timing mutations relative to WGD

Strict criteria were used to define a mutation as pre-WGD in a simulated ‘single biopsy’ analysis. Using data from a single region, we inferred whether a variant’s copy number status tracked the major or minor copy number allele. For example, with LOH (that is, minor copy number is 0), the presence of a variant means it must track with the major allele. Where the variant copy number is larger than the minor copy number, it too must track the major allele. Where there is only one major and/or one minor copy of the allele, we cannot infer whether the variant occurred pre- or post-WGD and we categorized these timings as ‘unclear’. Where the variant copy number is less than or equal to the minor copy number, we assume it is tracked with the minor allele. To minimize false categorization of variants as pre-WGD, we performed a proportion test using the mutation’s VAF and used the estimated 95% lower limit VAF to calculate the minimum copy number state for the variant, and used this to define the timing of the variant relative to WGD. Similarly, to minimize falsely categorizing variants as post-WGD, we used the estimated 95% upper limit VAF to calculate the maximum copy number state for the variant and inferred the timing. If the classification of the variant differed when using the upper limit and lower limits, the timing was then defined as ‘unclear’. Where there are two WGD events in a single region, this method times the variant relative to the first WGD event. Thus, when describing variants as ‘pre’ or ‘post’ WGD, we refer to the first WGD event.

For the driver mutation analysis, we leveraged evidence from all regions in the tumour as well as using the maximum copy number state for the variant, calculating from the 95% upper confidence interval from the VAF proportion test to avoid falsely categorizing an event as occurring post-WGD. Where there is subclonal WGD, the presence of a variant in a non-WGD region suggests that the event must have occurred pre-WGD.

### HLA LOH prediction

HLA LOH prediction for the sequenced regions was performed using LOHHLA^27^.

### Peptide vaccine design and manufacture

The vaccine was manufactured by the GMP & T Cell Therapy core facility (German Cancer Research Centre, DKFZ, Heidelberg, Germany) in accordance with facility standard operating procedures, using variant data from the sequenced supraclavicular LN and the RUL lesion (available sequencing at time of manufacture). netMHCpan 4.0 (ref. ^49^) was used to predict the affinity of the peptides. Priority was given to variants that seemed clonal at that time/were present in both samples, and that had a high predicted affinity (less than 1,000 nM, and ideally less than 500 nM), resulting in 14 candidate targets (Extended Data Table 1). The exceptions to these criteria are the *TP53* p.P118L (lower affinity) and *EGFR* p.T790M (single sample), which were included because of clinical interest; and *GFPT1* p.L598V (lower affinity), which was found at high VAFs in both the supraclavicular LN and RUL lobe. Briefly, for the manufacturing, solid phase synthesis using Fmoc chemistry was applied in a fully automated multiple synthesizer (Syro II, MultiSynTech). Synthesis was carried out on preloaded Wang-resins with 2-(1H-Benzotriazole-1-yl)-1,1,3,3-tetramethyluronium hexafluorophosphate (HBTU) as a coupling agent. More than 5,000 peptides have been manufactured at this facility for research purposes. Quality control checks are in place to safeguard against contamination and ensure correctness of the sequence. The 14 candidate peptides (24–29 amino acids in length) were dissolved in water with 10% DMSO for infusion, and four peptides were found to be insoluble (*GFPT1* p.L580V, *KLHL26* p.R190W, *SLC27A4* p.T329M, *EGFR* p.T790M). The remaining ten long peptides were used for injection. Each vaccine contained 60 µg in 60 µl per peptide (10 peptides at 600 µl total), and mixed with 600 µl of Montanide ISA 51 to formulate the vaccine. Pooled peptides were injected intradermally.

### ctDNA analyses

Patient-specific anchored-multiplex PCR enrichment panels were generated using 467 autosomal somatic mutations detected from the tissue WES output^20^. Additionally, mutations in genes associated with resistance to *EGFR* TKI therapy were also explored: these include mutations in *PIK3CA*, *KRAS*, *NRAS*, *BRAF* and *EGFR* (Supplementary Table 2). Libraries were prepared according to the ArcherDX LiquidPlex ctDNA protocol for Illumina with the following modifications: the first PCR was performed using these cycling conditions: 95 °C for 3 min, 11 cycles: 95 °C for 30 s and 65 °C for 15 min, followed by 72 °C for 3 min and a hold at 4 °C. The second PCR was performed using these cycling conditions: initial denaturation at 95 °C for 3 min, 15 cycles: 95 °C for 30 s and 65 °C for 15 min, followed by 72 °C for 3 min and a hold at 4 °C. Libraries were sequenced on an Illumina NextSeq sequencer to approximately 50 million read pairs per sample and the resulting FASTQs were analysed using the Archer Analysis circulating free DNA variant calling pipeline^20^. Copy number aberrations associated with resistance were explored from low pass whole genome sequencing of the circulating free DNA using ichorCNA (v.0.1.0)^50^.

### RNA-seq sample sequencing and bioinformatics pipeline

The extraction and sequencing pipelines are summarized in a previous TRACERx manuscript^34^. Danaher gene signatures^29^ and CIBERSORTx^30^ were used to deconvolute the immune microenvironment.

### FISH

FISH was carried out using the Vysis EGFR/CEP7 FISH Probe set (Abbott Molecular) in combination with the Histology FISH Accessory Kit (Agilent Technologies). Freshly cut 4-µM pathology sections were xylene-dewaxed, followed by serial rehydration into FISH buffer. Sections were incubated at 98 °C for 10 min in hybridization pre-treatment solution followed by on-section pepsin digestion for 10 min at 37 °C. After serial dehydration, FISH probes were applied to the section, sealed with rubber cement glue and co-denatured at 71 °C for 5 min. Probe/tissue annealing for 16 h was followed by a 65 °C stringent wash for 10 min. Sections were dehydrated, antifade mounting media containing DAPI (Vectashield) was applied and then sections were visualized using a Zeiss Observer Z1 microscope.

### Immune analyses

#### Tissue culture

Blood samples were collected in Vacutainer EDTA blood collection tubes (BD) and PBMCs isolated within 24 h of apheresis by density gradient centrifugation (750*g* for 10 min) on Ficoll Paque Plus (GE Healthcare). The interface was washed twice with complete RPMI-1640, and cells were resuspended in 90% FBS with 10% DMSO (Sigma-Aldrich) and cryopreserved in liquid nitrogen.

#### MANAFEST assay

PBMCs were thawed, washed and seeded at 200,000 cells per well in a 96-well plate, in duplicate, in TexMACS media (Miltenyi) containing 5% human AB serum, penicillin-streptomycin and amphotericin B (all from Sigma-Aldrich), and 10 ng ml^−1^ human interleukin (IL)-15 plus 50 ng ml^−1^ human IL-21 (all cytokines from BioLegend), with IL-2 (Proleukin, Clinigen) added on day 1 at a final concentration of 40 IU ml^−1^. Cells were maintained in culture with regular feeding or passage as required, every 2–3 days, in 5% CO_2_ at 37 °C for a total of 11 days. 24–29-mer neopeptides were synthesized by a manufacturing process that achieves purity of 95%, in which all peptides showed one major peak at the expected molecular weight (Pepscan/Biosynth). Lyophilized peptides were reconstituted in ultra-pure DMSO (Sigma-Aldrich) and added on day 0 at a final concentration of 1 µg ml^−1^. A cocktail of 8–12-mer viral peptides derived from human CMV, EBV and flu was used as a positive control (Peptivator, Miltenyibiotec) and added at day 0 at a final concentration of 1 µg ml^−1^ for each peptide. Cell pellets were collected on day 11 and lysate stored in RLT Buffer at −80 °C before RNA extraction (RNAeasy Mini kit, Qiagen). The number of detectable TCRs at baseline was set to 1 from 0 for fold change visualization for which no response was detected. Ex vivo T cell receptor sequencing (TCR-seq) repertoires were isolated from thawed PBMCs cultured overnight without cytokine stimulation.

#### TCR-seq

TCR alpha and beta sequencing was performed on RNA extracted from MANAFEST assay PBMC cultures and bulk RNA acquired from the RUL and SCLC-transformed liver metastasis, using a quantitative experimental and computational TCR-seq pipeline described recently^51,52^. This protocol incorporates a unique molecular identifier attached to each complementary DNA TCR molecule that enables correction for PCR and sequencing errors. The suite of tools used for TCR identification, error correction and CDR3 extraction are freely available at https://github.com/innate2adaptive/Decombinator.

#### TCR-seq analysis

The 3,000 most abundant unique beta chain CDR3s from each sample were selected for analysis as previously described^51^. Where multiple clones showed equal abundance at rank 3,000, the count value closest to 3,000 was used as a cut-off. Samples were analysed using backend code from the MANAFEST^23^ webtool (https://sourceforge.net/projects/manafest/; http://www.stat-apps.onc.jhmi.edu/FEST). Neopeptide-stimulated samples were analysed relative to cytokine alone control from the matched time point. Clones were classified as significantly enriched in a given condition if they exclusively showed an odd’s ratio > 10 and *Q* < 0.01 by false discovery rate-corrected Fisher’s exact test compared with the no peptide (cytokine only) control condition. Only clones present at 500 or more copies in the test condition were considered for analysis unless otherwise specified, with or without being detected in the control condition. Ex vivo samples yielded fewer than 3,000 unique TCR sequences and were not used for MANAFEST analysis. For visualization and calculation of fold change in the number of detected clones, a 0 value is ascribed a value of 1. Analysis was conducted in R using the dplyr (v.1.1.4), immunarch (v.0.9.1), data.table (v.1.14.8), RColorBrewer (v.1.1-3), viridis (v.0.6.5) and ggplot2 (v.3.5.1) packages.

#### TCR clustering through Gliph2

To identify groups of TCRs that shared similar sequence structure to the clones that were significantly expanded in PBMC samples from the MANAFEST assay, we clustered together the top 3,000 CDR3B sequences from each time point (months 30, 40, 45), from each condition (Cytokine, CEF, ex19del, T790m), using the Gliph2 clustering algorithm. Gliph2 generates output scores per cluster by quantifying clonal expansion and estimating the likelihood that those sequences will cluster together. More significant clusters with more unique TCR sequences are located towards the centre of the network plot, whereas clusters with weaker connections are located further out. To verify that the expanded sequences were driven by peptide-specific stimulation, we allocated TCR clusters to a condition using a 50% threshold to ensure each cluster was included only once in the analysis and we maximized all available data. We then compared cluster importance scores of cytokine culture alone with other conditions (CEF, ex19del and T790M). For Gliph2 TCR clustering, the top 3,000 CDR3B sequences with matching TRBV genes and a count of 3 or greater from the PBMC samples were clustered together using the ‘gliph2’ function from the turboGliph package^53^ (v.0.99.2). All productive CDR3B sequences with a corresponding V gene were included. Clusters were assigned to a condition on the basis of a count proportion threshold of more than 50%, so each cluster would be represented only once in the analysis. The cluster importance score is the −log_10_-transformed value of the ‘total.score’ metric from the Gliph2 output.

#### Fluorospot

PBMCs were thawed, washed and seeded at 5 × 10^6^ cells per well of a 24-well plate in TexMACS media (Miltenyi) containing 5% human AB serum (Sigma-Aldrich), penicillin-streptomycin (Sigma-Aldrich) and 10 ng ml^−1^ human IL-15 plus 50 ng ml^−1^ human IL-21 (both from BioLegend), with IL-2 added on day 2 at a final concentration 40 IU ml^−1^. A cocktail of all ten vaccine and four non-vaccine long neopeptides was added at a final concentration of 1 µg ml^−1^ on day 1. Cells were maintained in culture with regular feeding or passage as required, every 2–3 days, in 5% CO_2_ at 37 °C for a total of 11 days, before washing and re-plating overnight in media deprived of cytokine. Rested cells were plated for Fluorospot analysis at 150,000 cells per well and re-stimulated for 24 h with 1 µg ml^−1^ peptide, 2 µg ml^−1^ phytohaemagglutinin (Sigma-Aldrich) or anti-CD3 (1 µg ml^−1^), plus anti-CD28 (20 µg ml^−1^) antibodies supplied in the Fluorospot kit for Human GZMB and IFNG used as per the manufacturer’s instructions (MabTech). Plates were protected from light until being read on an AID iSPOT plate reader and analysed by automated spot counting.

### Reporting summary

Further information on research design is available in the Nature Portfolio Reporting Summary linked to this article.

## Online content

Any methods, additional references, Nature Portfolio reporting summaries, source data, extended data, supplementary information, acknowledgements, peer review information; details of author contributions and competing interests; and statements of data and code availability are available at 10.1038/s41586-025-08586-y.

## Supplementary information

Supplementary Information This supplementary information file contains the following sections: orthogonal phylogenetic trees, exploration of characteristics of samples with a high proportion of subclonal pre-whole genome doubling variants; and supplementary tables on sample characteristics, mechanisms of resistance to *EGFR* TKI explored using ctDNA, HLA class I prediction results, immune genes explored as potential mechanisms of immune evasion and *CXCL9* expression.

Reporting Summary

Supplementary Data This supplementary data file contains the CDR3B TCR sequence results from the MANAFEST assay.

## Figures and Tables

**Extended Data Table 1:**
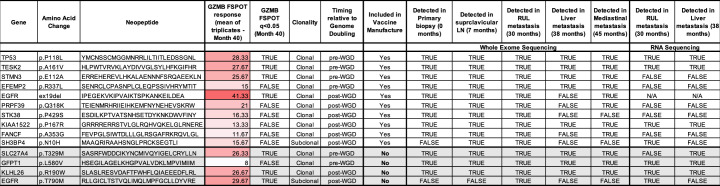
Neoepitope summary

Neoepitope peptides annotated with granzyme B (GZMB) response, clonality in cancer, timing relative to genome doubling, and presence in sequenced regions using whole exome sequencing and RNA sequencing. 4 peptides were insoluble and therefore not included in final vaccine production.

## Data Availability

The whole-exome sequencing and RNA sequencing data used in this manuscript have been deposited in the European Genome–phenome Archive (EGA: EGAS00001007926), which is hosted by the European Bioinformatics Institute (EBI) and the Centre for Genomic Regulation (CRG). All processed data files used to reproduce figures are available at *Zenodo* (10.5281/zenodo.14028323)^54^.

## Code availability

All code is available at *Zenodo* (10.5281/zenodo.14028323)^54^.
