# Supplementary material for: Clonal driver neoantigen loss under EGFR TKI and immune selection pressures
Source: Nature. 2025 Feb 19;639(8056):1052–9. doi: 10.1038/s41586-025-08586-y (PMC11946900; doi:10.1038/s41586-025-08586-y)
Supplement: Supplementary file 1 — This supplementary information file contains the following sections: orthogonal phylogenetic trees, exploration of characteristics of samples with a high proportion of subclonal pre-whole genome doubling variants; and supplementary tables on sample characteristics, mechanisms of resistance to EGFR TKI explored using ctDNA, HLA class I prediction results, immune genes explored as potential mechanisms of immune evasion and CXCL9 expression. [file 41586_2025_8586_MOESM1_ESM.docx]

**Clonal Driver Neoantigen Loss under *EGFR* TKI and Immune Selection Pressures**

**Supplementary Information**

**Al Bakir, Reading et al. 2024**

**Table of Contents**

[Supplementary Note 2](#_Toc189062892)

[Orthogonal phylogenetic analyses 2](#_Toc189062893)

[SciClone 2](#_Toc189062894)

[MEDICC2 2](#_Toc189062895)

[Exploring characteristics of samples with a high proportion of subclonal pre-whole genome doubling variants 3](#_Toc189062896)

[Supplementary Tables 4](#_Toc189062897)

[Sample characteristics 4](#_Toc189062898)

[Mechanisms of resistance to *EGFR* TKI explored using ctDNA 5](#_Toc189062899)

[HLA class I prediction results 6](#_Toc189062900)

[Immune genes 6](#_Toc189062901)

[*CXCL9* expression 6](#_Toc189062902)

[References 7](#_Toc189062903)

# Supplementary Note

## Orthogonal phylogenetic analyses

### SciClone

SciClone^55^ (v1.1.0) was also used to generate mutation clusters to compare to the output from the PyClone based method described in the case report (Supplementary Figure 1). SciClone clustering did not take into account copy number aberrations and clustered based on mutation variant allele frequencies (VAFs). With the exception of PyClone Cluster 1, each PyClone Cluster lies within a single sciClone cluster. The variation seen in PyClone cluster 1 is explained by copy number differences such as genome doubling status of the mutation or copy number losses. These results suggest that our PyClone clustering output is likely robust.


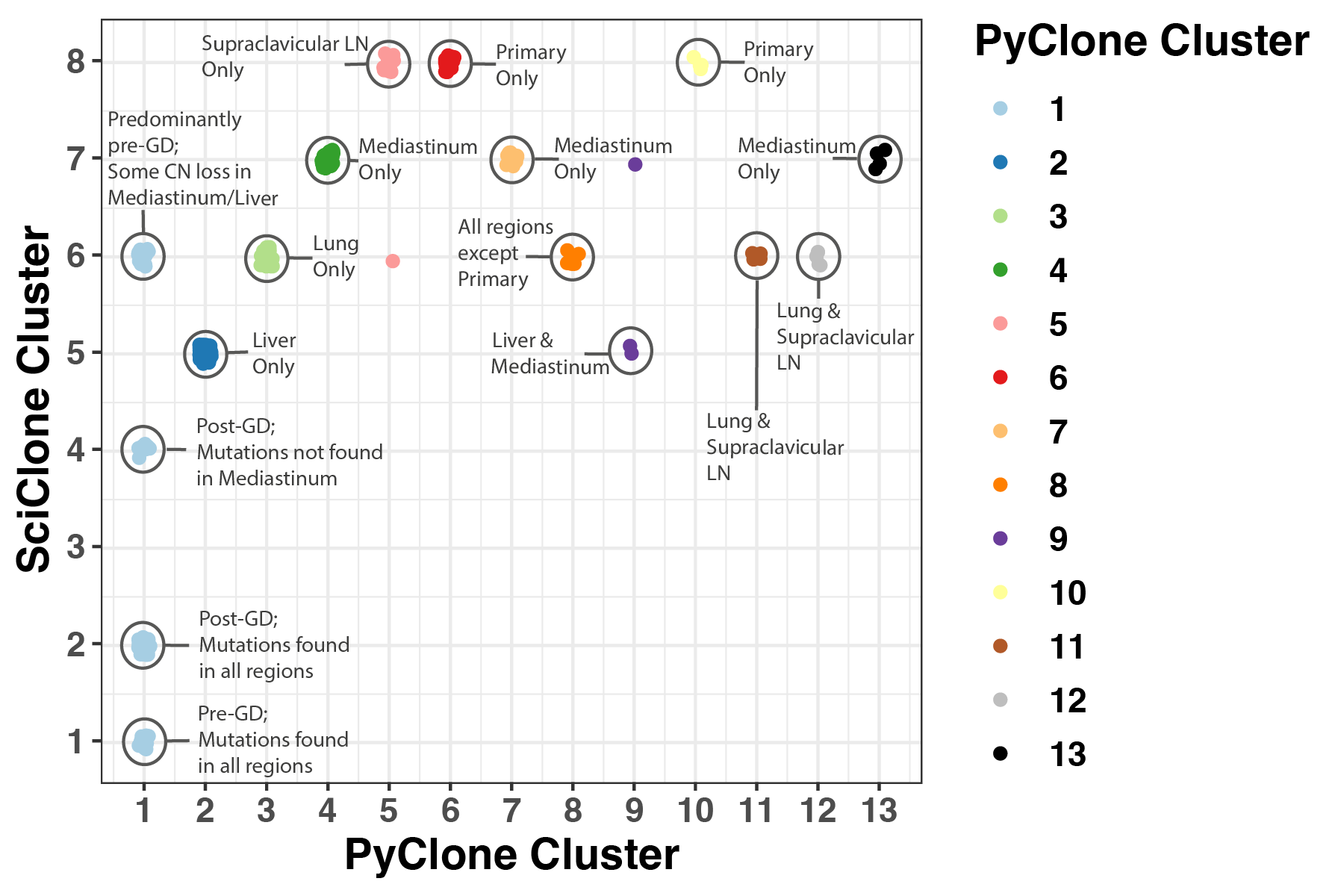


**Supplementary Figure 1.** Comparison of SciClone and PyClone clustering.

### MEDICC2

Regional somatic copy number trees were also generated to understand the relationship of the primary and metastatic regions using MEDICC2^56,57^ (Minimum Event Distance for Intra-tumour Copy number Comparisons, v1.3.2). Whilst this only provided a region-based tree (Supplementary Figure 2), it supports the finding that the liver metastasis (38 months) and the mediastinal mass (45 months) are more closely related to each other; and that the supraclavicular LN (7 months) and RUL metastasis (19 months) are more closely related to each other, supporting the phylogenetic tree generated (**Fig. 1b**).


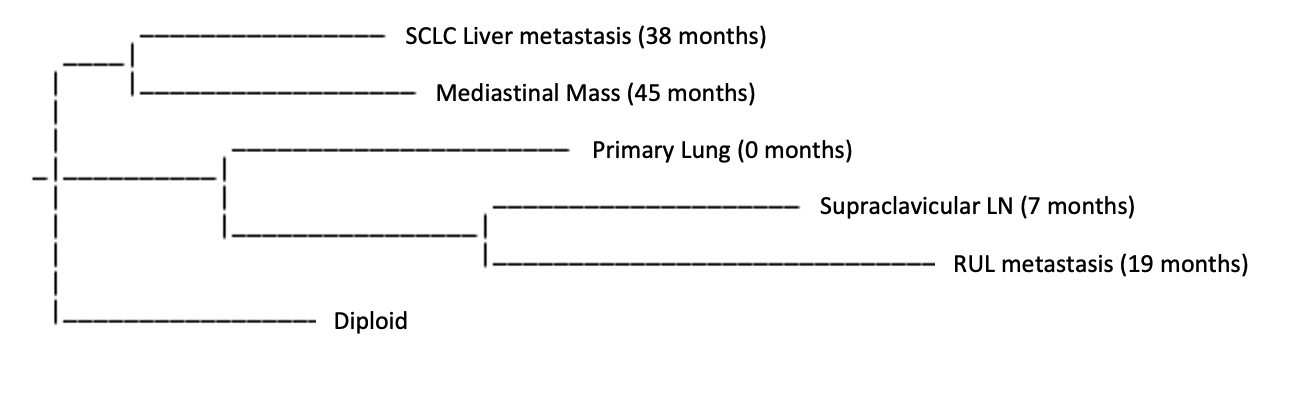


**Supplementary Figure 2.** MEDICC somatic copy number region-based tree

## Exploring characteristics of samples with a high proportion of subclonal pre-whole genome doubling variants

The characteristics of samples with a low proportion of clonal, pre-WGD mutations were compared to those with a high proportion of clonal, pre-WGD mutations (lowest 5th centile vs remaining samples). There was no difference in total number of mutations explored in each region (lowest vs remaining samples: 105 vs 161; Wilcoxon rank sum test *p*=0.14), number of genome doubling events detected in that region (Fisher’s exact test *p*=0.32), weighted genomic instability index of that region (wGII – a measure of chromosomal instability; lowest vs remaining samples: 0.57 vs 0.56, Wilcoxon rank sum test *p*=0.26) or histology (Fisher’s exact test *p*=0.66). Strikingly, regions with a high proportion of subclonal pre-WGD variants were enriched in tumours with subclonal WGD events and tumours with a higher number of sampled regions (*p*<2.2e-16 & *p*=0.045 respectively; ANOVA testing of fixed linear effects model incorporating tumour GD clonality, number of regions sequenced, histology, tumour size, region wGII and number of GD events in each region; **Fig. 3**).

#

# Supplementary Tables

##

## Sample characteristics


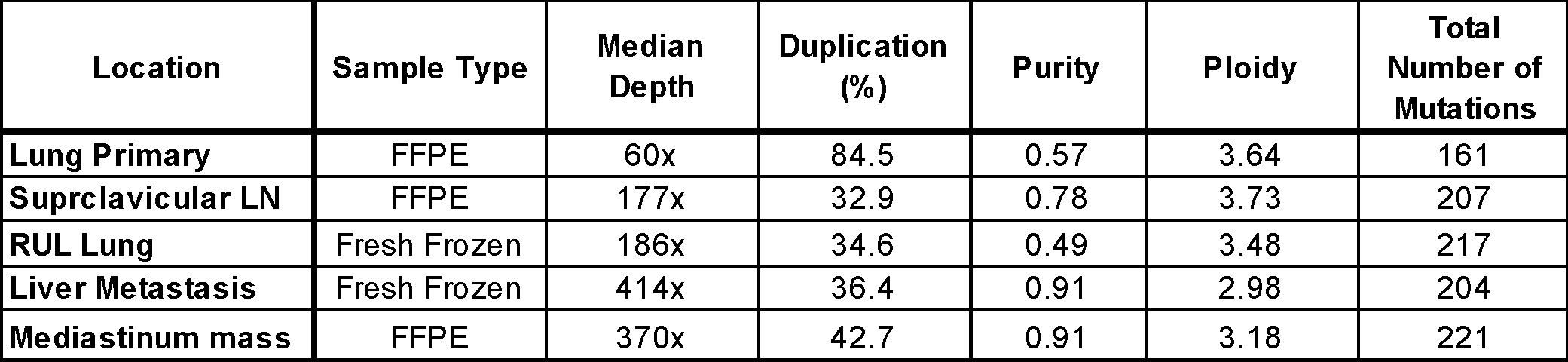


**Supplementary Table 1.** Summary of sample characteristics including sample type, depth, duplication, purity, ploidy, and total number of somatic mutations detected mutations

##

## Mechanisms of resistance to *EGFR* TKI explored using ctDNA

None of the mechanisms of resistance to *EGFR* TKI therapy highlighted in Supplementary Table 2 were identified in the ctDNA at any timepoint.


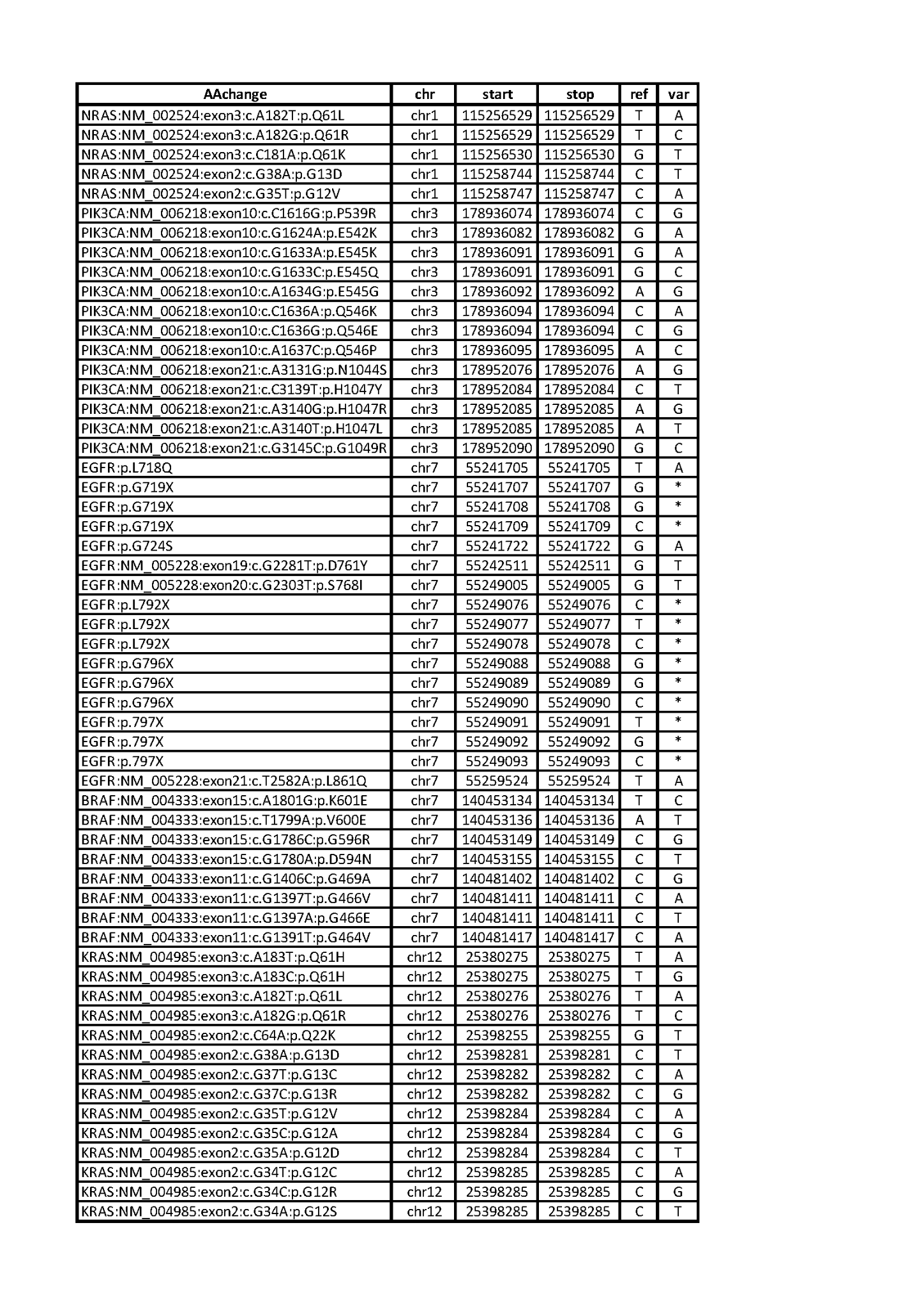


**Supplementary Table 2.**Point mutation mechanisms of resistance to *EGFR* TKIs were explored using ctDNA

## HLA class I prediction results

| **HLA I** | **Predicted HLA Typing** |
| --- | --- |
| **A** | HLA-A*23:01:01 |
|  | HLA-A*03:01:01:01 |
| **B** | HLA-B*44:03:01 |
|  | HLA-B*07:02:01 |
| **C** | HLA-C*07:02:01:03 |
|  | HLA-C*04:01:01:01 |

**Supplementary Table 3.** Human Leukocyte Antigen (HLA) Class I prediction results

## Immune genes

Genomic mechanisms of immune evasion were explored in the genomic data using the gene list summarised in Supplementary Table 4. There were no variants identified in the whole exome sequencing data for all regions sequenced.


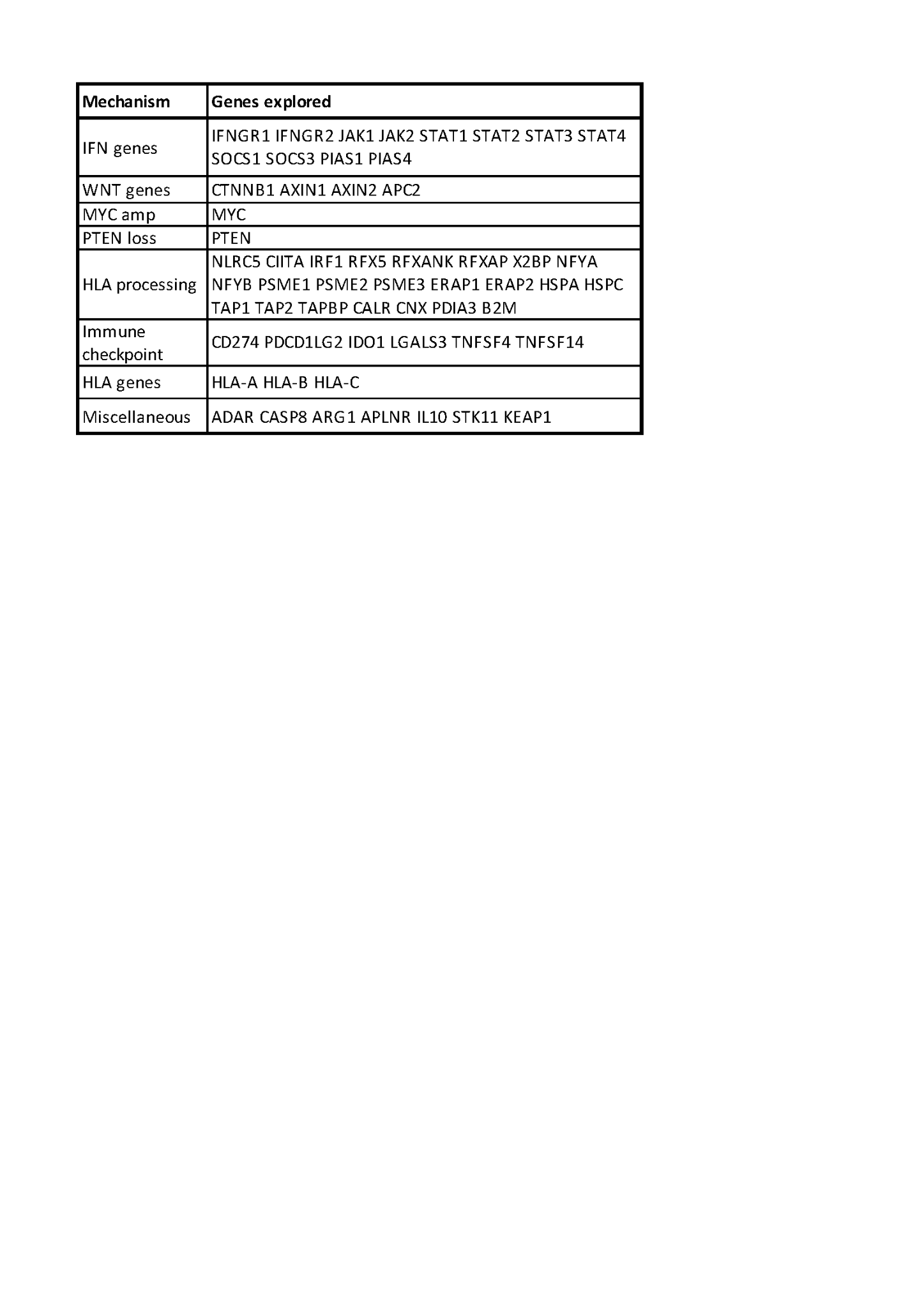


**Supplementary Table 4.** List of genes associated with immune evasion.

## *CXCL9* expression

*CXCL9* expression was compared in the RUL metastasis, SCLC-transformed liver metastasis & in the TRACERx 421 *EGFR*-mutant NSCLC cohort (54 regions in 23 patients)


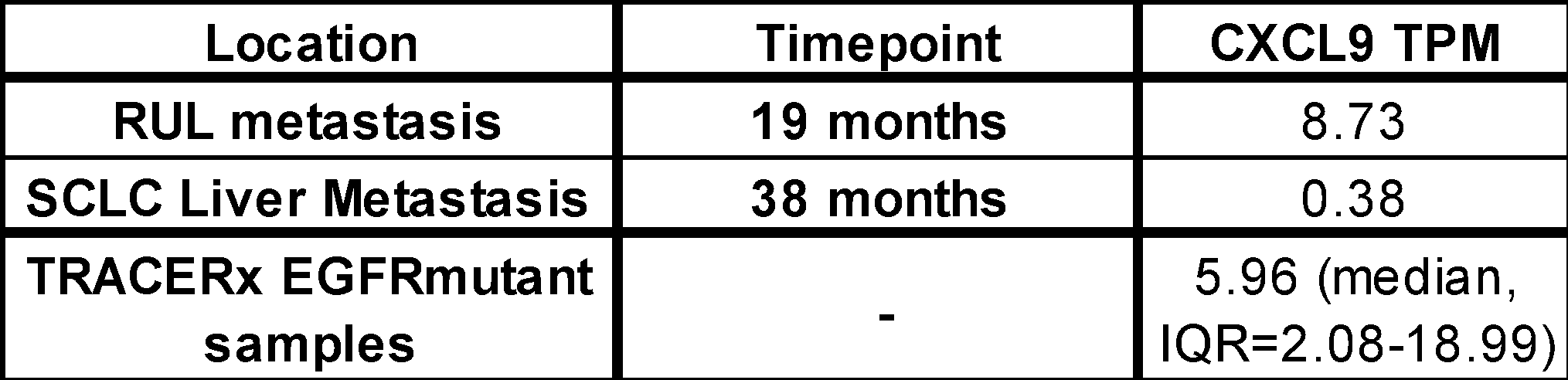


**Supplementary Table 5.** *CXCL9* TPM in the RUL & Liver metastases, as well as in the TRACERx 421 EGFR-mutant NSCLC cohort (54 regions in 23 patients)

# References

55. [Miller, C. A. *et al.* SciClone: inferring clonal architecture and tracking the spatial and temporal patterns of tumor evolution. *PLoS Comput. Biol.* **10**, e1003665 (2014).](http://paperpile.com/b/JMPLip/BrQc)

56. [Schwarz, R. F. *et al.* Phylogenetic quantification of intra-tumour heterogeneity. *PLoS Comput. Biol.* **10**, e1003535 (2014).](http://paperpile.com/b/JMPLip/j9nC)

57. [Kaufmann, T. L. *et al.* MEDICC2: whole-genome doubling aware copy-number phylogenies for cancer evolution. *Genome Biol.* **23**, 241 (2022).](http://paperpile.com/b/JMPLip/ynqr)
