# Supplementary material for: Clonal driver neoantigen loss under EGFR TKI and immune selection pressures
Source: Nature. 2025 Feb 19;639(8056):1052–9. doi: 10.1038/s41586-025-08586-y (PMC11946900; doi:10.1038/s41586-025-08586-y)
Supplement: Supplementary file 2 — Reporting Summary [file 41586_2025_8586_MOESM2_ESM.pdf]

nature portfolio

Corresponding author(s): Charles Swanton, Sergio Quezada, Nicholas McGranahan

Last updated by author(s): Nov 7, 2024

Reporting Summary

Nature Portfolio wishes to improve the reproducibility of the work that we publish. This form provides structure for consistency and transparency in reporting. For further information on Nature Portfolio policies, see our Editorial Policies and the Editorial Policy Checklist.

Statistics

For all statistical analyses, confirm that the following items are present in the figure legend, table legend, main text, or Methods section.

n/a	Confirmed	
☐	☒	The exact sample size (*n*) for each experimental group/condition, given as a discrete number and unit of measurement
☒	☐	A statement on whether measurements were taken from distinct samples or whether the same sample was measured repeatedly
☐	☒	The statistical test(s) used AND whether they are one- or two-sided<br>*Only common tests should be described solely by name; describe more complex techniques in the Methods section.*
☒	☐	A description of all covariates tested
☒	☐	A description of any assumptions or corrections, such as tests of normality and adjustment for multiple comparisons
☒	☐	A full description of the statistical parameters including central tendency (e.g. means) or other basic estimates (e.g. regression coefficient) AND variation (e.g. standard deviation) or associated estimates of uncertainty (e.g. confidence intervals)
☒	☐	For null hypothesis testing, the test statistic (e.g. *F*, *t*, *r*) with confidence intervals, effect sizes, degrees of freedom and *P* value noted<br>*Give P values as exact values whenever suitable.*
☒	☐	For Bayesian analysis, information on the choice of priors and Markov chain Monte Carlo settings
☒	☐	For hierarchical and complex designs, identification of the appropriate level for tests and full reporting of outcomes
☒	☐	Estimates of effect sizes (e.g. Cohen's *d*, Pearson's *r*), indicating how they were calculated

Our web collection on statistics for biologists contains articles on many of the points above.

Software and code

Policy information about availability of computer code

Data collection	NoNo software was used
Data analysis	Analysis was conducted in R (4.2.1) using the dplyr (v1.1.4), immunarch (v0.9.1), data.table (v1.14.8), RColorBrewer (v1.1-3), viridis (v0.6.5), ggplot2 (v3.5.1), fst (v0.9.8), stringr (v1.4.1), ggpubr(v0.4.0), gridExtra(v2.3), xlsx(v0.6.5), tidyr(v1.2.1), ggbeeswarm(0.7.1), lme4(v1.1-35.1), reshape2(v1.4.4), microViz(v0.11.0), ggridges(v0.5.4), scales(v1.2.1), phangorn(v2.11.1), heatplus(v3.4.0), ape(v5.6-2), and gplots(v3.1.3) packages.<br><br>The code and data to generate all the figures is available on Zenodo: https://doi.org/10.5281/zenodo.14028323

For manuscripts utilizing custom algorithms or software that are central to the research but not yet described in published literature, software must be made available to editors and reviewers. We strongly encourage code deposition in a community repository (e.g. GitHub). See the Nature Portfolio guidelines for submitting code & software for further information.

Data

Policy information about availability of data

All manuscripts must include a data availability statement. This statement should provide the following information, where applicable:

- Accession codes, unique identifiers, or web links for publicly available datasets
- A description of any restrictions on data availability
- For clinical datasets or third party data, please ensure that the statement adheres to our policy

The Whole Exome Sequencing and RNA Sequencing data used in this manuscript has been deposited in the European Genome–phenome Archive (EGA: EGAS00001007926), which is hosted by The European Bioinformatics Institute (EBI) and the Centre for Genomic Regulation (CRG).

Research involving human participants, their data, or biological material

Policy information about studies with human participants or human data. See also policy information about sex, gender (identity/presentation), and sexual orientation and race, ethnicity and racism.

Reporting on sex and gender	Not applicable, single case report
Reporting on race, ethnicity, or other socially relevant groupings	Not applicable, single case report
Population characteristics	Not applicable, single case report
Recruitment	Not applicable, single case report
Ethics oversight	Clinical oversight of the vaccine therapy was undertaken at University Medical Centre, Heidelberg. The patient was treated with a personalised peptide vaccine within the scope of an individual healing attempt [statement WD 9 – 3000 – 083/23 of the German Parliament, guidelines 2001/20/EG and 2005/28/EG, Declaration of Helsinki of the World Medical Association (Article 37)]; approval by the institutional review board and ethics committees is not required. Informed consent for genetic and immune research studies was obtained in accordance with protocols approved by the University Medical Centre Heidelberg institutional review board. Written informed consent to transfer and perform analyses at the Francis Crick Institute and associated institutions was also provided.

Note that full information on the approval of the study protocol must also be provided in the manuscript.

Field-specific reporting

Please select the one below that is the best fit for your research. If you are not sure, read the appropriate sections before making your selection.

☒ Life sciences ☐ Behavioural & social sciences ☐ Ecological, evolutionary & environmental sciences

For a reference copy of the document with all sections, see nature.com/documents/nr-reporting-summary-flat.pdf

Life sciences study design

All studies must disclose on these points even when the disclosure is negative.

Sample size	Not applicable, single case report
Data exclusions	Not applicable, single case report
Replication	Not applicable, single case report
Randomization	Not applicable, single case report
Blinding	Not applicable, single case report

Reporting for specific materials, systems and methods

We require information from authors about some types of materials, experimental systems and methods used in many studies. Here, indicate whether each material, system or method listed is relevant to your study. If you are not sure if a list item applies to your research, read the appropriate section before selecting a response.

Materials & experimental systems

n/a	Involved in the study
☒	☐ Antibodies
☒	☐ Eukaryotic cell lines
☒	☐ Palaeontology and archaeology
☒	☐ Animals and other organisms
☐	☒ Clinical data
☒	☐ Dual use research of concern
☒	☐ Plants

Methods

n/a	Involved in the study
☒	☐ ChIP-seq
☒	☐ Flow cytometry
☒	☐ MRI-based neuroimaging

Clinical data

Policy information about clinical studies

All manuscripts should comply with the ICMJE guidelines for publication of clinical research and a completed CONSORT checklist must be included with all submissions.

Clinical trial registration: Not applicable as this is a descriptive case study

Study protocol: Not applicable as this is a descriptive case study

Data collection: Not applicable as this is a descriptive case study

Outcomes: Not applicable as this is a descriptive case study

Plants

Seed stocks: Not applicable as this is a descriptive case study

Novel plant genotypes: Not applicable as this is a descriptive case study

Authentication: Not applicable as this is a descriptive case study
